# Cooperative inhibition of SNARE-mediated vesicle fusion by α-synuclein monomers and oligomers

**DOI:** 10.1038/s41598-021-90503-0

**Published:** 2021-05-26

**Authors:** Gyeongji Yoo, Sanghun Yeou, Jung Bae Son, Yeon-Kyun Shin, Nam Ki Lee

**Affiliations:** 1 School of Interdisciplinary Bioscience and Bioengineering, Pohang University of Science and Technology, Pohang, 37673 Korea; 2 Department of Physics, Pohang University of Science and Technology, Pohang, 37673 Korea; 3 Department of Chemistry, Seoul National University, Seoul, 08826 Korea; 4 Roy J. Carver Department of Biochemistry, Biophysics and Molecular Biology, Iowa State University, Ames, IA 50011 USA

**Keywords:** Molecular neuroscience, Synaptic vesicle exocytosis

## Abstract

The primary hallmark of Parkinson's disease (PD) is the generation of Lewy bodies of which major component is α-synuclein (α-Syn). Because of increasing evidence of the fundamental roles of α-Syn oligomers in disease progression, α-Syn oligomers have become potential targets for therapeutic interventions for PD. One of the potential toxicities of α-Syn oligomers is their inhibition of SNARE-mediated vesicle fusion by specifically interacting with vesicle-SNARE protein synaptobrevin-2 (Syb2), which hampers dopamine release. Here, we show that α-Syn monomers and oligomers cooperatively inhibit neuronal SNARE-mediated vesicle fusion. α-Syn monomers at submicromolar concentrations increase the fusion inhibition by α-Syn oligomers. This cooperative pathological effect stems from the synergically enhanced vesicle clustering. Based on this cooperative inhibition mechanism, we reverse the fusion inhibitory effect of α-Syn oligomers using small peptide fragments. The small peptide fragments, derivatives of α-Syn, block the binding of α-Syn oligomers to Syb2 and dramatically reverse the toxicity of α-Syn oligomers in vesicle fusion. Our findings demonstrate a new strategy for therapeutic intervention in PD and related diseases based on this specific interaction of α-Syn.

## Introduction

α-Synuclein (α-Syn), an abundant presynaptic protein, is a major component of the amyloid fibrils called Lewy bodies which are the primary hallmark of a variety of neurodegenerative diseases, such as Parkinson’s disease (PD), dementia with Lewy bodies, and other synucleinopathies^1–5^. α-Syn is a natively unfolded protein that misfolds into β-sheet-rich amyloid-like fibrils under pathological condition, generating intermediate oligomeric species^6,7^. Oligomers or protofibrils of α-Syn are regarded as toxic species leading to neuronal death^6–10^. Soluble high-molecular-weight oligomers of α-Syn were found in the brain tissue of patients with PD^11,12^, and oligomerization was accelerated by autosomal familial PD mutations of α-Syn^7,12,13^. Animal and cellular models support a pathogenic role of α-Syn oligomers in diseases^14–16^. Due to increasing evidence of the fundamental roles of α-Syn oligomers in disease progression, the oligomeric forms of α-Syn have emerged as one of the most compelling therapeutic targets for PD and related neurodegenerative disorders^15,17–20^. It has been challenging, however, to develop effective strategies to suppress oligomerization of α-Syn and its associated toxicity because the assembly of the oligomers is dependent on multiple factors, including the concentration of the protein, oxidative stress, heavy metal, pH, and temperature in cells^21–24^. Moreover, the molecular mechanisms underlying how α-Syn oligomers drive the death of neurons remain unclear^17^. A deeper understanding of the pathology of α-Syn oligomers is required for developing improved therapeutic strategies.

Although the physiological function of the α-Syn monomer is still controversial, α-Syn, enriched in presynaptic terminals, plays an important role in SNARE-mediated synaptic vesicle trafficking and neurotransmission^25–29^. Südhof and his coworkers reported that α-Syn directly binds to the N-terminal domain of synaptobrevin-2 (Syb2), a vesicular soluble *N*-ethylmaleimide-sensitive factor attachment protein receptor (v-SNARE) protein in synaptic vesicles^26^. The specific interaction between α-Syn and Syb2 helps the assembly of the SNARE complex^26^ and induces the clustering of vesicles or Syb2, which promotes SNARE complex formation^25,30^.

Because the physiological roles of α-Syn are correlated with synaptic transmission, the toxicity of α-Syn oligomers very likely also stems from the physiological functions of the α-Syn monomer in synaptic transmission. Indeed, the interactions between α-Syn and Syb2 are preserved in dopamine-induced α-Syn oligomers^15^, but the effects of the oligomers are completely opposite; for α-Syn oligomers, the α-Syn–Syb2 interaction interferes with SNARE complex formation^15^, whereas the α-Syn monomer promotes SNARE complex formation using the same interaction with Syb2^25,26^. In addition to an in vitro study, α-Syn oligomers generated by dopamine in vivo induce nigrostriatal degeneration and associated symptoms^18^.

**Figure 1:**
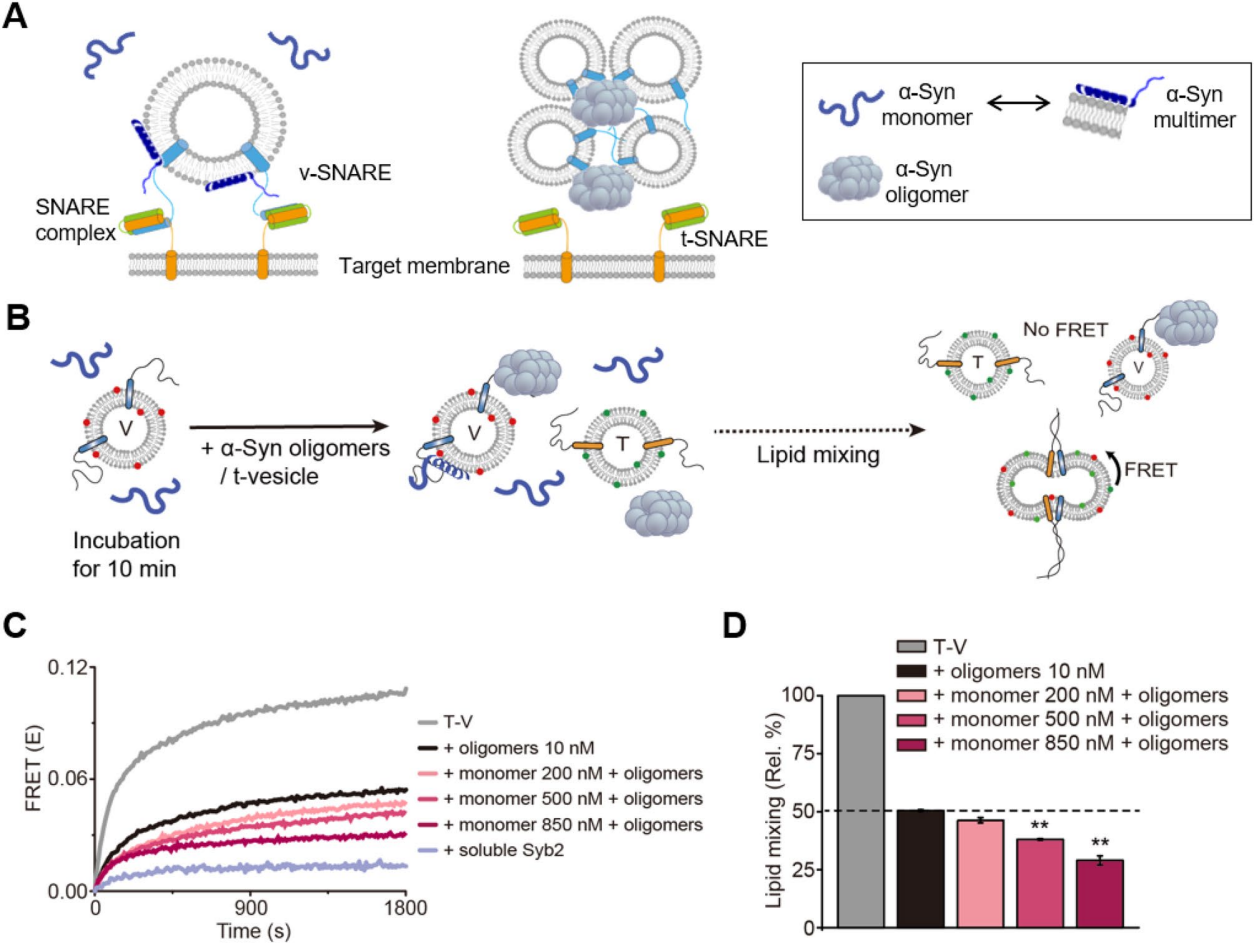
Cooperative inhibition of SNARE-mediated membrane fusion by α-Syn monomer and oligomers. (**A**) Mechanistic models of α-Syn monomer and oligomer actions in SNARE-mediated membrane fusion. (Left panel) α-Syn multimers bind to the N-terminal domain of vesicular SNARE (v-SNARE) using its C-terminal domain, which promotes SNARE complex formation. (Right panel) Using the same binding mode, α-Syn oligomers inhibit SNARE-mediated vesicle fusion. (**B**) Scheme of the in vitro lipid-mixing assay. v-Vesicles (V) were incubated with α-Syn monomer for 10 min, and then oligomers and t-vesicles (T) were added to the premixture. These Figures were produced by Adobe illustrator CS6 (Adobe, San Jose, California, USA) (https://www.adobe.com/kr/products/illustrator.html) and edited by Microsoft Powerpoint professional plus 2019 (Microsoft, Redmond, Washington, USA). (**C**) Cooperative inhibition of vesicle fusion by α-Syn monomer and oligomers (a representative graph showing a FRET change). T and V (20 μM lipid concentration) were mixed together at 37 °C without α-Syn (T-V, gray line). With the addition of α-Syn oligomers (10 nM), a significant reduction in fusion efficiency was observed (the black line). When α-Syn monomer was added to the T–V mixture together with oligomers, the FRET signal was lower than that of the α-Syn oligomer-treated vesicle fusion. Lipid mixing was confirmed to be driven by SNARE complex formation by the addition of the soluble domain of Syb2, which blocked lipid mixing (the light purple line). (**D**) Relative percentages of lipid mixing at 1800s from the FRET data in (**C**) (± SEM, N = 3; three measurements were performed in different days). **P < 0.01 by ANOVA test. The graphs shown were produced using Originlab 8.5 software (OriginLab Corporation, Northhampton, MA, USA) (https://www.originlab.com/index.aspx?go = PRODUCTS/Origin) and edited by Adobe Illustrator CS6 (Adobe).

Since α-Syn monomers and oligomers, binding in the same mode to the N-terminal domain of Syb2, showed opposite functions in neuronal SNARE-mediated vesicle fusion^15,25,26^ (Fig. 1A), a question arises: what happens to SNARE-mediated vesicle fusion when α-Syn oligomers, inhibiting vesicle fusion, and α-Syn monomers, which promote vesicle fusion, come together? As α-Syn oligomers are mixed with a greater amount of monomeric α-Syn in the presynaptic terminals, investigating the inhibitory effect of α-Syn oligomers on vesicle fusion in the presence of α-Syn monomers is a prerequisite for improving our understanding of the toxicity of α-Syn oligomers.

Here, we show that the α-Syn monomer and oligomers cooperatively inhibit SNARE-mediated vesicle fusion. This cooperative effect results from the clustering of Syb2-carrying vesicles. Thus, the fusion inhibitory effect of α-Syn oligomers should be more severe in the presynaptic region where the α-Syn monomer is enriched. Based on this cooperative inhibition mechanism, we designed a strategy to reverse the fusion inhibitory effect of α-Syn oligomers. We reversed the fusion inhibition of α-Syn oligomers by modulating the interactions between α-Syn oligomers and Syb2 using small peptide fragments of α-Syn. The small peptide fragments compete with α-Syn oligomers for binding to Syb2 and dramatically restore SNARE-mediated vesicle fusion in the presence of α-Syn oligomers. Our findings suggest a new strategy for therapeutic intervention in PD and related diseases based on this specific protein–protein interaction of α-Syn.

## Results

### The α-Syn monomer enhances the toxicity of dopamine-induced α-Syn oligomers in inhibiting SNARE-mediated vesicle fusion

When α-Syn oligomers and monomers are added to the reaction mixture for vesicle fusion, three scenarios are possible (Supplementary Fig. S1A): (1) α-Syn monomer binds to Syb2 more weakly than α-Syn oligomers do. Thus, the α-Syn monomer has no effect on the fusion inhibitory effect of α-Syn oligomers. (2) Both the α-Syn monomer and oligomers interact with Syb2, competing with each other for Syb2 binding. As a result, the addition of the monomer attenuates the fusion inhibitory effect of oligomers. (3) α-Syn monomers and oligomers cooperate, and monomer addition thus increases fusion inhibition toxicity.

To address this question, we performed an in vitro lipid-mixing assay using fluorescently labeled proteoliposomes^31,32^. In this assay, t-SNAREs [complexes of syntaxin H_abc_-trunctated (HT) (amino acids 168–288 of syntaxin 1A, lacking the Habc domain) and synaptosomal-associated protein-25 (SNAP25)] and v-SNARE Syb2 were incorporated into two different liposomes (t- and v-vesicles, respectively)^15,31^. When t-vesicles doped with donor dyes (T) and v-vesicles doped with acceptor dyes (V) were fused together through SNARE complex formation^31,33,34^, the lipids from the two types of vesicles mixed (Fig. 1B). This lipid mixing was monitored by the fluorescence resonance energy transfer (FRET) signal between 1,1′-dioctadecyl-3,3,3′,3′-tetramethylindocarbocyanine perchlorate (DiI) and 1,1′-dioctadecyl3,3,3′,3′-tetramethylindodicarbocyanine perchlorate (DiD) dyes (Supplementary Fig. S1B). α-Syn oligomers were prepared by incubating the α-Syn monomer (15 μM) with dopamine (100 μM) at 37 °C for 72 h (Supplementary Fig. S2A) and purified using size exclusion chromatography (Supplementary Fig. S2B and C)^15,35–37^. We have estimated that the oligomers contain approximately 17 α-Syn monomeric subunits in our previous work^15^. Without the addition of α-Syn oligomers, the T-V mixture presented a significant mixing of lipids (Fig. 1C, the gray line). We confirmed that lipid mixing was driven by SNARE complex formation, as the addition of the soluble domain of Syb2 blocked lipid mixing (Fig. 1C, the light purple line).

Then, we applied α-Syn oligomers to the T-V reaction mixture. When we used 10 nM α-Syn oligomers, the extent of lipid mixing was reduced by approximately 50% compared with that of the control (Fig. 1C, the black line), which is consistent with the results of our previous work^15^. Next, we incubated V with α-Syn monomer for 10 min, and then α-Syn oligomers and T were added to the premixture (Fig. 1B). To our surprise, more severe inhibition of vesicle fusion between T and V was observed as the concentration of α-Syn monomer increased (Fig. 1C). When 10 nM oligomers were added together with 850 nM monomer, the fusion efficiency was reduced to 25%; the 25% fusion efficiency was almost the same fusion efficiency when 30 nM oligomers were used (Fig. 1D)^15^. As a control, α-Syn monomer at submicromolar concentrations (200–850 nM) was shown to have a negligible effect on lipid mixing (Supplementary Fig. S3). This result suggests that even a small amount of α-Syn oligomers would cause severe toxicity by inhibiting synaptic vesicle fusion in presynaptic regions where the α-Syn monomer is abundant.

### α-Syn monomers and oligomers cooperatively induce the clustering of synaptic vesicle mimics

Next, we investigated how the α-Syn monomer enhances the fusion inhibitory effect of dopamine-induced α-Syn oligomers. We first tested whether the incubation of the α-Syn monomer with oligomers increases the amount of α-Syn oligomers. When we incubated the α-Syn monomer with preformed oligomers for 12 h without dopamine, no change in the amounts of oligomers and monomers was observed (Supplementary Fig. S4). This is consistent with previous work showing that dopamine-modified α-Syn oligomers are not seeding competent^18^.

**Figure 2:**
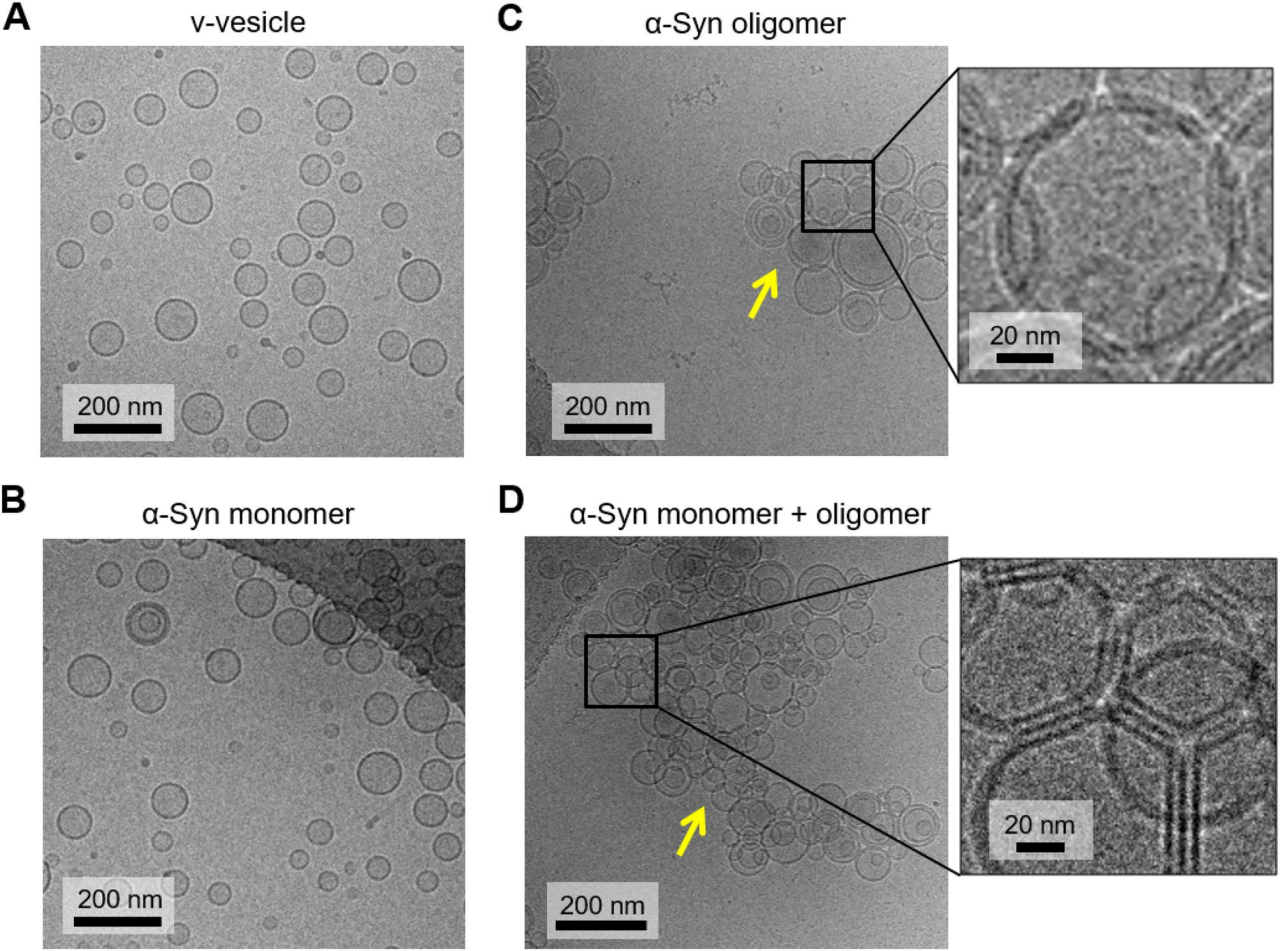
Clustering of v-vesicles by α-Syn oligomers. (**A**) Representative image of v-vesicles without α-Syn. (**B**) v-Vesicles in the presence of α-Syn monomer. The molar ratio of lipid/α-Syn monomers was 10:1 (5 μM: 0.5 μM.) The dark region is the holey carbon grid. (**C**) v-Vesicles in the presence of α-Syn oligomers. The molar ratio of lipid/α-Syn oligomers was 200:1. Clustering of v-vesicles was observed (the yellow arrowhead). (**D**) v-Vesicles treated with both α-Syn monomer and oligomers. The molar ratio of lipid/α-Syn oligomers/α-Syn monomers was 200:1:20. The yellow arrowhead points to large clusters of the vesicles. The vesicle cluster was significantly larger than that observed in (**C**). The enlarged image shows the locally flat and thickened adhesion regions between vesicles, which are not observed in (**C**). All images were edited by Microsoft Powerpoint professional plus 2019 (Microsoft).

Then, we tested the effect on vesicle clustering. We have proposed that α-Syn oligomers inhibit vesicle fusion by clustering synaptic vesicle mimics in vitro, thereby limiting the number of vesicles available for docking^15^. A few micromolar α-Syn monomer is also known to induce vesicle clustering^27,29,30,38^. Thus, it is possible that the α-Syn monomer by itself cannot induce vesicle clustering at the low concentration (less than 1 μM) that we applied in this work but assists α-Syn oligomers in clustering vesicles. We obtained images of v-vesicles treated with α-Syn oligomers in the presence and absence of monomers using cryo-electron microscopy (cryo-EM) (Fig. 2). The molar ratio of lipid: α-Syn oligomers: α-Syn monomers was 200:1:20. When more than ten vesicles were attached together, we assigned them as a vesicle clustering. In the absence of α-Syn, v-vesicles appeared without clustering (Fig. 2A). When we added the α-Syn monomer, no clustering of v-vesicles was observed (Fig. 2B). However, in the presence of α-Syn oligomers, v-vesicles formed compact clusters with irregular shapes (Fig. 2C). Cryo-EM could not resolve α-Syn oligomers because of its resolution limit. Since α-Syn oligomers are known to inhibit vesicle fusion through binding to the N-terminal domain of Syb2^15^, we tested if their specific interactions with Syb2 are responsible for clustering the vesicles. We reconstituted v-vesicles with Syb2 lacking the N-terminal region (nt-Syb2, amino acids 29–116) and incubated them with α-Syn oligomers. No clustering of v-vesicles was observed by cryo-EM (Supplementary Fig. S5). In addition, protein-free vesicles were not clustered by α-Syn oligomers (Supplementary Fig. S5). These results show that α-Syn oligomers cluster vesicles using their specific interactions with Syb2. When v-vesicles were incubated with both α-Syn monomer and oligomers, they formed larger clusters (Fig. 2D) than those shown in Fig. 2C. While 22.7 ± 1.2 vesicles consisted of a cluster with α-Syn oligomers, 123.5 ± 11.5 vesicles clustered after the addition of α-Syn monomers together with oligomers. Interestingly, when the α-Syn monomer was added with oligomers to v-vesicles, locally flat and thickened adhesion regions between vesicles were often visualized (Fig. 2D, enlarged image). Although it is difficult to quantify the fraction of the flattened region, this structure was not observed in images of vesicles treated with α-Syn oligomers only (Fig. 2C, enlarged image). The addition of α-Syn monomer with α-Syn oligomers seemed to induce tighter adhesion between the vesicles. These results suggest that the combination of the α-Syn monomer and oligomers cooperatively clusters Syb2-carrying vesicles and that the α-Syn monomer may act as an adhesive between the vesicles.

### Membrane-bound α-Syn monomers are required for cooperative fusion inhibition

**Figure 3:**
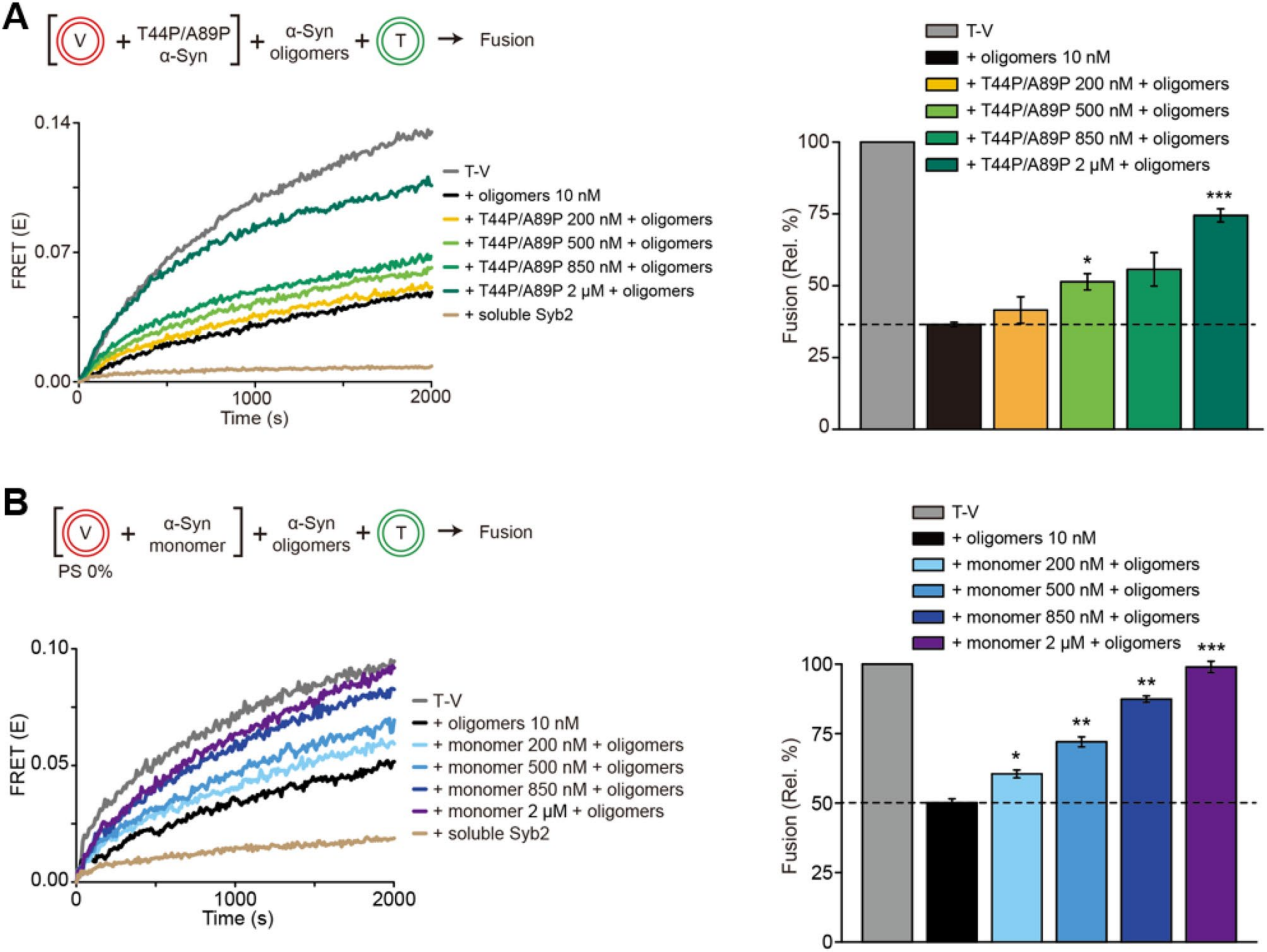
α-Syn monomer interactions with the lipid membrane enable cooperative inhibition of vesicle fusion. (**A**) The effect of the lipid-binding-deficient mutant of the α-Syn monomer (T44P/A89P) on fusion inhibition. v-Vesicles (V) were incubated with the T44P/A89P monomer for 10 min, and then α-Syn oligomers and t-vesicles (T) were added to the premixture. T and V (20 μM lipid concentration) were mixed together at 37 °C without α-Syn (T-V, gray line). Fusion efficiency was significantly reduced by the addition of α-Syn oligomers (10 nM, the black line). When the T44P/A89P α-Syn monomer was added to the T–V mixture together with oligomers, the FRET signal increased (200 nM, yellow line; 500 nM, light green line; 850 nM, green line; 2 μM, dark green line). A control measurement using soluble Syb2 confirmed that lipid mixing was driven by SNARE complex formation (beige line). Bar graphs were obtained from the values at 2000s from the FRET measurements (± SEM, N = 3). *P < 0.05, **P < 0.01, ***P < 0.001 by Student’s t test. (**B**) The effect of the negatively charged lipid (PS) on fusion inhibition. v-Vesicles (V) prepared with 0% PS were used in the measurement. v-Vesicles without PS were incubated with wild-type α-Syn monomer, and then t-vesicles (T) and oligomers were added to the premixture. The gray and black lines denote the T–V mixture and the T–V mixture with α-Syn oligomers, respectively. When wild-type α-Syn monomer was added to the T–V mixtures together with oligomers, the FRET signal increased (200 nM, cyan line; 500 nM, light blue line; 850 nM, navy line; 2 μM, purple line). A control measurement using soluble Syb2 confirmed that lipid mixing was driven by SNARE complex formation (the beige line). Bar graphs were obtained from the values at 2000s from the FRET measurements (± SEM, N = 3). *P < 0.05, **P < 0.01, ***P < 0.001 by Student’s t test. All graphs were produced using OriginLab 8.5 software (OriginLab Corporation) and edited by Adobe Illustrator CS6 (Adobe).

Next, we investigated the mechanism whereby α-Syn monomers and oligomers cooperatively cluster synaptic vesicle mimics and inhibit SNARE-mediated vesicle fusion at the molecular level. It is well known that the α-Syn monomer binds to negatively charged phospholipids^25,30,39^. Thus, we examined whether the interactions between the α-Syn monomer and the negatively charged membrane play a role in increasing the fusion inhibitory effect of α-Syn oligomers. We prepared the T44P/A89P mutant of α-Syn, which retains the ability to bind Syb2 but has a reduced lipid-binding affinity^39^. We measured the effect of this mutant on lipid mixing using the in vitro lipid-mixing assay (Fig. 3A). When we added both monomeric T44P/A89P mutant and α-Syn oligomers, the cooperative fusion inhibitory effect of α-Syn oligomers and wild-type α-Syn monomer was not observed. Surprisingly, in contrast to the case of the wild-type α-Syn monomer, vesicle fusion became more active as the concentration of the T44P/A89P mutant increased (Fig. 3A). As a control, when 850 nM T44P/A89P was added to the fusion mixture in the absence of α-Syn oligomers, the extent of lipid mixing was nearly invariant, but it slightly decreased at 2 μM T44P/A89P (Supplementary Fig. S5). This result indicates that the binding of the α-Syn monomer to the lipid membrane is required for the cooperative inhibition of vesicle fusion by the α-Syn monomer and oligomers.

To confirm the role of membrane binding by the α-Syn monomer in cooperative fusion inhibition, we removed negatively charged 1,2-dioleoyl-sn-glycero3-(phospho-l-serine) (PS) from v-vesicles (Fig. 3B). We used the wild-type α-Syn monomer in this measurement. As shown in Fig. 3B, the cooperative fusion inhibitory effect disappeared again. The fusion inhibition by α-Syn oligomers was attenuated by the addition of the α-Syn monomer (Fig. 3B), which was consistent with the results of adding the T44P/A89P mutant (Fig. 3A). Strikingly, the addition of 2 μΜ α-Syn monomer to the fusion mixture in the presence of α-Syn oligomers recovered almost 100% of the SNARE-mediated lipid mixing (Fig. 3B). These results clearly demonstrate that the binding of the α-Syn monomer to the membrane lipids is required for the cooperative fusion inhibition by α-Syn oligomers.

### Lipid-binding-deficient α-Syn mutants reduce the clustering of synaptic vesicle mimics by α-Syn oligomers

**Figure 4:**
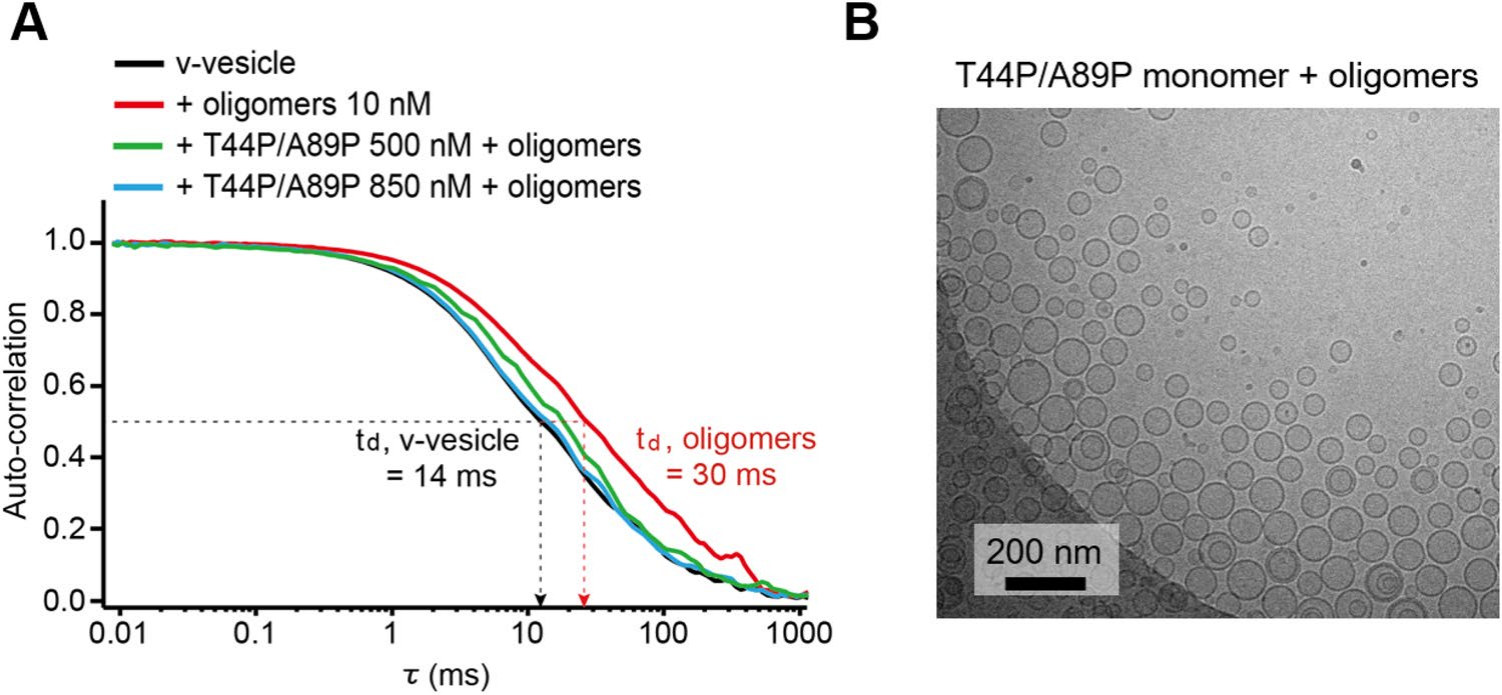
Lipid-binding-deficient α-Syn (T44P/A89P) reduces v-vesicle clustering induced by α-Syn oligomers. (**A**) Representative autocorrelation curves obtained by fluorescence correlation spectroscopy. The diffusion time (t_d_ = τ at G = 0.5) denotes the average transit time of vesicles through the confocal volume (Supplementary Fig. S6). The average diffusion times of v-vesicles, v-vesicles with α-Syn oligomers, v-vesicles with α-Syn oligomers and 500 nM T44P/A89P, and v-vesicles with α-Syn oligomers and 850 nM T44P/A89P were 14.1 ± 0.2 ms, 31.6 ± 0.8 ms, 20.8 ± 0.2 ms, and 15.1 ± 0.3 ms, respectively (± SEM, N = 3). The graph shown was produced using OriginLab 8.5 software (OriginLab Corporation) and edited by Adobe Illustrator CS6 (Adobe). (**B**) Cryo-electron microscopy image of v-vesicles with T44P/A89P and α-Syn oligomers. When more than ten vesicles were attached together, we assigned them as a vesicle clustering. v-Vesicles appeared mostly without clustering. The image was edited by Microsoft Powerpoint professional plus 2019 (Microsoft).

We showed that when the α-Syn monomer was treated with α-Syn oligomers, the wild-type α-Syn monomer induced clustering of v-vesicles and further inhibited vesicle fusion (Fig. 2D). In contrast to the wild-type α-Syn monomer, the α-Syn mutant T44P/A89P, having less lipid binding affinity, showed the opposite effect: the mutant attenuated the fusion inhibitory effect of α-Syn oligomers (Fig. 3A). We speculated that T44P/A89P α-Syn might alter the clustering of v-vesicles induced by α-Syn oligomers. To investigate this speculation quantitatively, we performed fluorescence correlation spectroscopy (FCS) measurements (Supplementary Fig. S6A). FCS measures the diffusion time (t_d_) of vesicles, which is reflected in an autocorrelation curve (Fig. 4A). The diffusion time is related to the diffusion coefficient, which is determined by the viscosity of the solvent and the hydrodynamic radius of the vesicles in solution^40^. When vesicles are clustered, their diffusion time increases (Supplementary Fig. S6B). The average diffusion time of v-vesicles was approximately 14 ms (Fig. 4A, black curve). The diffusion time of v-vesicles increased to 31 ms with the addition of α-Syn oligomers (Fig. 4A, red curve), which indicates that α-Syn oligomers induced clustering of v-vesicles. Since the diffusion time increased by approximately two-fold, the addition of α-Syn oligomers induces roughly ten times bigger vesicle clusters in average. When 500 nM and 850 nM monomeric T44P/A89P α-Syn were preincubated with v-vesicles and α-Syn oligomers were then added, the diffusion time of v-vesicles was reduced to 21 ms and 15 ms, respectively (Fig. 4A). These results indicate that T44P/A89P α-Syn attenuates the clustering of v-vesicles induced by α-Syn oligomers. The clustered vesicles have more chance to be deposited on the surface of the glass, while small vesicles are diffusing in solution and may be detected more frequently in FCS measurement. Thus, the diffusion times in Fig. 4A have to be used qualitatively for observing the effect of T44P/A89P α-Syn. To confirm the results of the FCS study in Fig. 4A, we obtained cyro-EM images of vesicles. The interpretation of Fig. 4A was further supported by cryo-EM images, showing that the addition of T44P/A89P α-Syn significantly reduces the clustering of v-vesicles (Fig. 4B) compared with the clustering in the α-Syn oligomer treatment in Fig. 2C. Taken together, these results clearly demonstrate that monomeric α-Syn binds to negatively charged lipids for helping the clustering of v-vesicles by α-Syn oligomers.

### Small peptide fragments of α-Syn reverse the fusion inhibitory effect of α-Syn oligomers

We showed that the T44P/A89P α-Syn monomer blocks the clustering of v-vesicles induced by α-Syn oligomers, which results in restoration of SNARE-mediated vesicle fusion. T44P/A89P α-Syn does not bind to membrane lipids but can interact with Syb2^39^. In the same manner, the dopamine-induced α-Syn oligomers do not interact with the lipid membrane but do interact with the N-terminal domain of Syb2, which results in the inhibition of SNARE-mediated lipid mixing. Considering that T44P/A89P α-Syn and α-Syn oligomers commonly interact with Syb2, it is possible that T44P/A89P α-Syn competes with α-Syn oligomers for binding to Syb2 and reduces the clustering of v-vesicles by α-Syn oligomers. This may restore the vesicle fusion in Fig. 3A. Similarly, when we used v-vesicles without PS (negatively charged lipid), wild-type α-Syn monomer could not bind to the vesicle membrane. Wild-type α-Syn monomer, however, could bind to Syb2, which restored vesicle fusion in the presence of α-Syn oligomers. These results strongly indicate that a competitor of α-Syn oligomer binding to Syb2 may reverse the fusion inhibitory effect of α-Syn oligomers.

**Figure 5:**
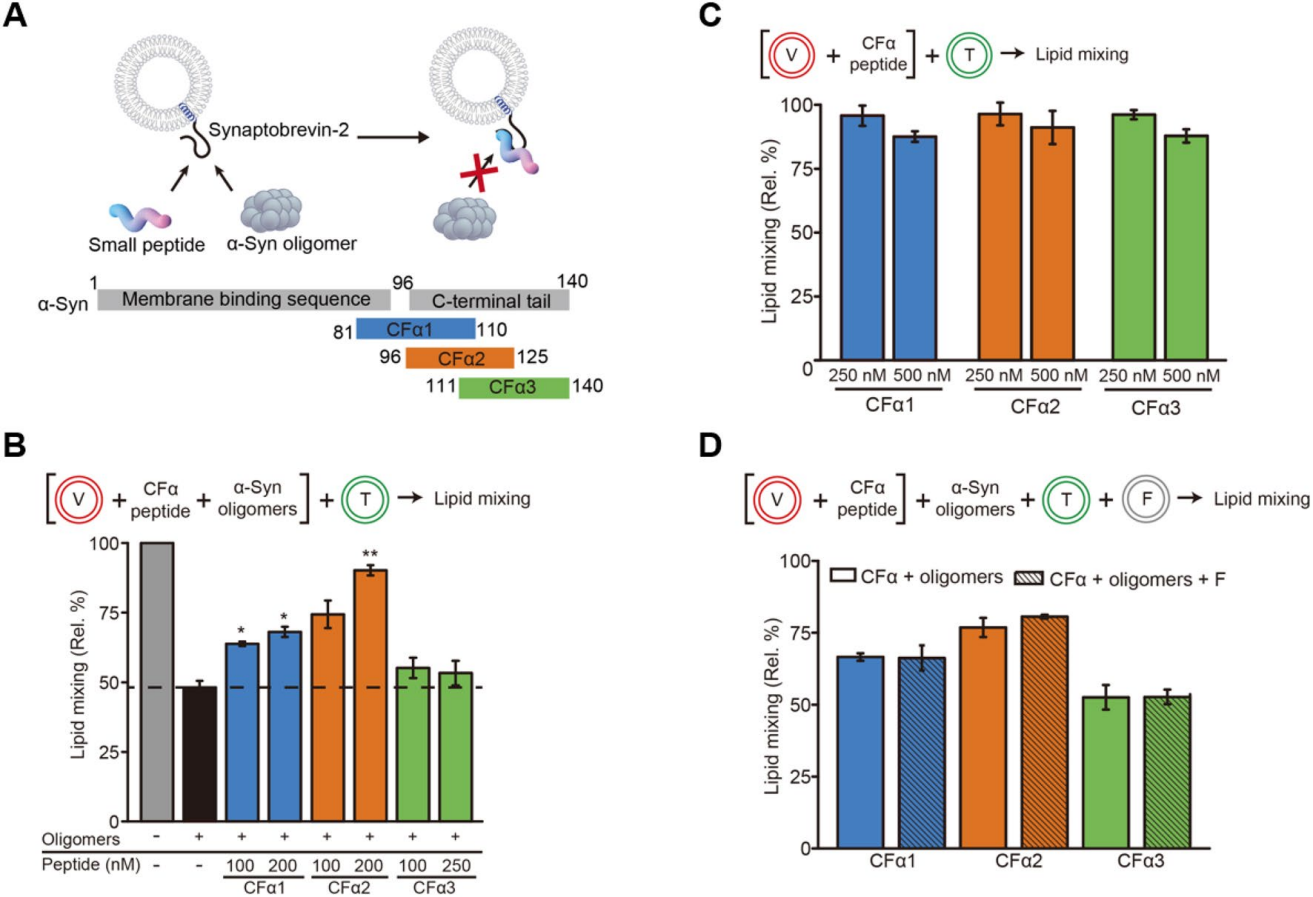
Reversal of α-Syn oligomer inhibition of fusion using small peptide fragments of α-Syn. (**A**) Thirty-residue peptides derived from the C-terminal domain of α-Syn, which bind to Syb2, competitively with α-Syn oligomers: CFα1 (a.a. 81–110), CFα2 (a.a. 96–125), and CFα3 (a.a. 111–140). This figure was created by Adobe illustrator CS6 (Adobe). (**B**) Small peptide-induced reversal of fusion inhibition by α-Syn oligomers. The dotted line indicates the extent of lipid mixing with α-Syn oligomers (10 nM). CFα1 and CFα2 increased vesicle fusion in the presence of α-Syn oligomers (10 nM). CFα3 had no effect on fusion inhibition by α-Syn oligomers (± SEM, N = 3). *P < 0.05, **P < 0.01, ***P < 0.001 by Student’s t test. (**C**) The effect of small peptides on vesicle fusion in the absence of α-Syn oligomers. CFα peptides (250 nM) showed a negligible effect on vesicle fusion, while 500 nM CFα peptides slightly reduced vesicle fusion (± SEM, N = 3). (**D**) Test of peptide binding to the lipid membranes. Protein-free vesicles (20 μM, **F**) were added to a 20 μM T-V mixture in the presence of small peptides (500 nM) and α-Syn oligomers (10 nM). The addition of the protein-free vesicles showed no effect on the reversal effect of the small peptides (± SEM, N = 3). All graphs shown were produced using OriginLab 8.5 software (OriginLab Corporation) and edited by Adobe Illustrator CS6 (Adobe).

To verify this, we set to reverse the fusion inhibitory effect of α-Syn oligomers using small peptide fragments that compete with the α-Syn oligomer for Syb2 binding and may be potential therapeutic agents (Fig. 5A). We prepared three 30-residue peptides (CFα1, CFα2, and CFα3) that were mainly derived from the C-terminal domain of α-Syn (Anygen, South Korea) (Fig. 5A; Table 1) because the C-terminal domain of α-Syn (a.a. 96–140) is known to bind to the N-terminal domain of Syb2^26^. We confirmed the binding between the peptide and Syb2 using fluorescence anisotropy assay (Supplementary Fig. S8). Next, v-vesicles were incubated with various concentrations of each peptide for 10 min in the presence of α-Syn oligomers (10 nM), and then t-vesicles were added into the premixture. We tested the effect of each peptide at the concentrations from 100 to 500 nM (Supplementary Figs. S9, S10, and S11). CFα1 and CFα2 effectively reversed the fusion inhibitory effect of α-Syn oligomers: 200 nM CFα1 increased the fusion efficiency from 45 to 68% (blue bars in Fig. 5B; Supplementary Fig. S9), and 200 nM CFα2 increased the fusion efficiency from 45 to 90% (orange bars in Fig. 5B; Supplementary Fig. S10). However, CFα3 had no effect on vesicle fusion in the presence of α-Syn oligomers (green bars in Fig. 5B; Supplementary Fig. S11). These results imply that the small peptide-induced reversal of fusion inhibition is sequence dependent, and thus, a specific interaction between small peptides and Syb2 is required for the reversal. The 96–125 C-terminal region of α-Syn seems to be used mostly for binding to Syb2, consistent with previous work suggesting that α-Syn 96–110 is the key domain for Syb2 binding^38^. For control experiments, no change in lipid mixing was observed with 250 nM peptides in the absence of α-Syn oligomers, while 500 nM peptides slightly reduced lipid mixing (Fig. 5C). In order to be used as clinical drugs, it needs to be tested whether the small peptides have any effect on the functions of α-Syn monomer by interfering the interactions between α-Syn monomer and Syb2 in animal models. At least, however, we showed that the small peptides do not interfere the SNARE-mediated vesicle fusion (Fig. 5C). In the presence of 500 nM α-Syn monomers, no change in the SNARE-mediated vesicle fusion was observed for in vitro FRET assays (Supplementary Fig. S12).

**Table 1:** Peptides derived from the C-terminal domain of α-Syn.

Peptide	Derived from	Sequence
CFα1	α-Syn 81–110	TVEGAGSIAAATGFVKKDQLGKNEEGAPQE
CFα2	α-Syn 96–125	KKDQLGKNEEGAPQEGILEDMPVDPDNEAY
CFα3	α-Syn 111–140	GILEDMPVDPDNEAYEMPSEEGYQDYEPEA

Then, we tested whether the small peptides bind to the lipid membrane. When we added 20 μM protein-free liposomes (F) to a 20 μM T-V mixture, no change in the reversal effect of CFα1 and CFα2 was observed (Fig. 5D). Dopamine-induced α-Syn oligomers do not bind to lipid membranes^15^. Thus, this result indicates that the CFα peptides, as expected, do not bind to lipid membranes (Fig. 5D). All these results indicate that the small peptides of CFα1 and CFα2 reverse the fusion inhibitory effect of α-Syn oligomers by disrupting the specific protein–protein interactions between α-Syn oligomers and Syb2.

## Discussion

**Figure 6:**
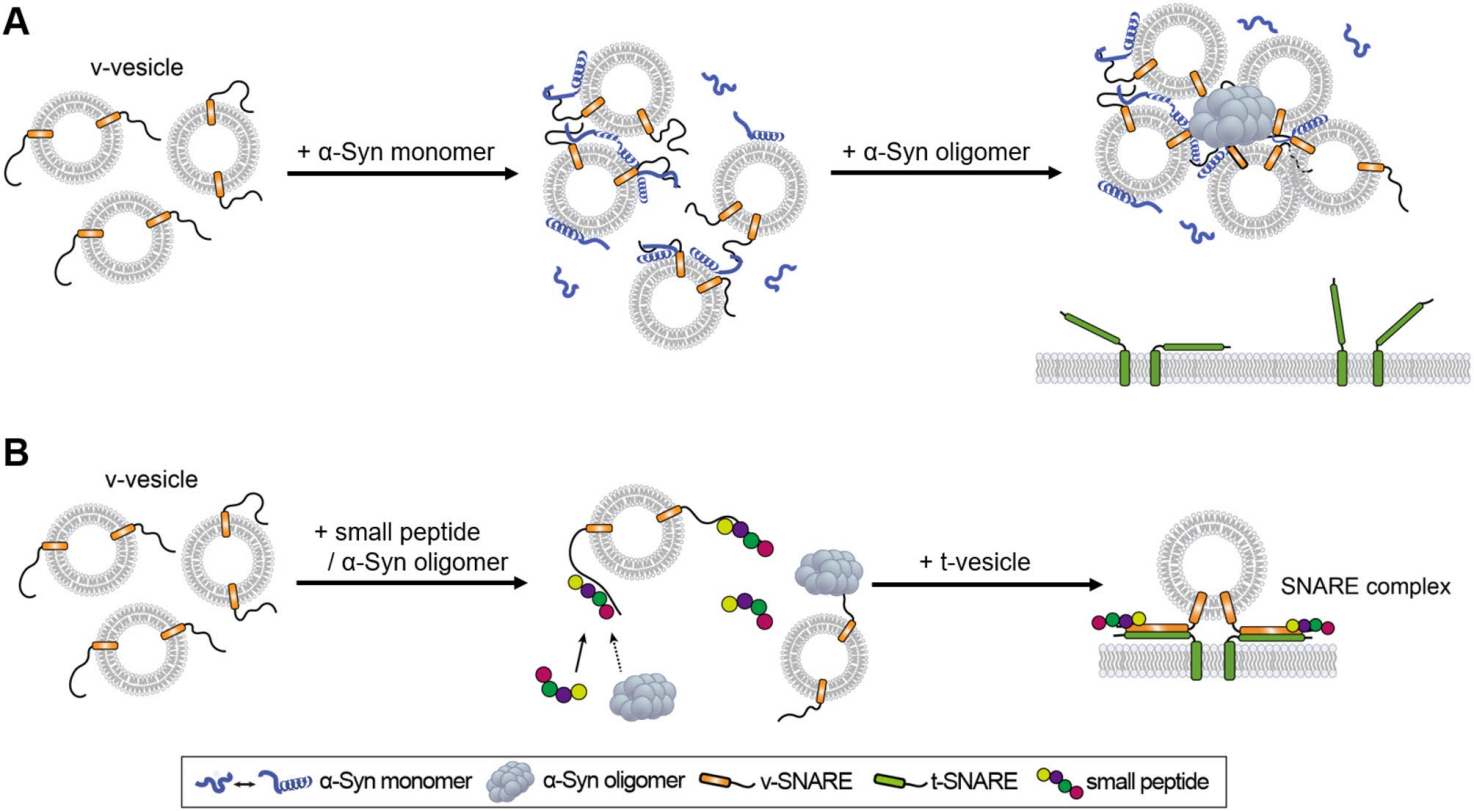
Proposed models for the cooperative inhibition of vesicle fusion by α-Syn monomer and oligomers and the reversal of α-Syn oligomer fusion inhibition. (**A**) The α-Syn monomer binds to lipid membranes and Syb2, which may induce a low degree of vesicle clustering^29,30,38,41^. α-Syn oligomers, which bind to Syb2, generate large clusters of v-vesicles with the help of the α-Syn monomer, which inhibits vesicle fusion. (**B**) Small peptide fragments derived from the C-terminal domain of α-Syn compete with α-Syn oligomers in binding Syb2. As a result, α-Syn oligomers cannot bind to Syb2 and induce the clustering of v-vesicles, which restores the formation of the SNARE complex and vesicle fusion. These figures were generated by Adobe Illustrator CS6 (Adobe).

In this work, we investigated the role of the interactions between α-Syn and v-SNARE Syb2 in the mechanism underlying α-Syn oligomer toxicity in vesicle fusion. We propose a mechanistic model for the cooperative inhibition of SNARE-mediated vesicle fusion by α-Syn monomer and oligomers (Fig. 6A). The α-Syn monomer simultaneously binds to the membrane lipid using its amphipathic N-terminal region and to Syb2 using its C-terminal region^25,29,38^. Recently, Fusco et al. showed that α-Syn monomer clusters vesicles using its two lipid binding sites^41^. Thus, the α-Syn monomer may induce a low degree of vesicle clustering^29,30,38,41^, but this clustering does not interfere with vesicle fusion. α-Syn oligomers, however, induce severe clustering of vesicles^15^. In the presence of α-Syn oligomers, the membrane lipid- and Syb2-bound α-Syn monomer assists the formation of large vesicle clusters, which results in severe fusion inhibition (Fig. 6A).

In our previous work, we reported that α-Syn oligomers effectively inhibit SNARE-mediated vesicle docking^15^. For the molecular mechanism of the inhibition, we proposed that α-Syn oligomers interfere with vesicle docking by clustering vesicles. In the present work, we clearly showed, by using cryo-EM, that α-Syn oligomers indeed cluster Syb2-carrying vesicles (Fig. 2). Moreover, we showed that the clustering of vesicles can be reversed by blocking the interactions between α-Syn oligomers and Syb2 (Figs. 5, 6B). This clearly demonstrates that α-Syn oligomers induce vesicle clustering using their specific interactions with Syb2 (Fig. 6A). We note that the soluble α-Syn oligomers in this work was generated by dopamine oxidation. Other α-Syn oligomers need to be investigated to generalize our finding.

Burre et al*.* reported that α-Syn multimers (or oligomers) generated upon membrane binding promote SNARE complex assembly^25^. Notably, these α-Syn multimers bound to the membrane are normal assemblies that become monomers when they are released to the cytosol. However, α-Syn oligomers generated by dopamine as in this work, which are abnormal aggregates, have a low binding affinity to membranes^15,35^ and are very stable in the cytosol^35–37^. In addition to the in vitro work, Mor et al*.* recently showed that dopamine increases the level of α-Syn oligomers and induces progressive nigrostriatal degeneration in vivo^18^.

Nonaggregated α-Syn is enriched in presynaptic terminals; its cellular concentration is estimated to be 6 μM^42,43^. Nonaggregated α-Syn or its monomer uses its interaction with Syb2 to help SNARE complex assembly^26^ and induces the clustering of vesicles or Syb2, which promotes SNARE complex formation^25,30^. Recently, a nonaggregated α-Syn monomer was reported to be beneficial for SNARE-dependent vesicle docking even at the excessive concentration 10 μΜ^42^. All the functions of the α-Syn monomer are beneficial for neurotransmitter release. However, our results show that the generation of α-Syn oligomers, although low in abundance, incapacitates the potential benefit of the α-Syn monomer at the physiologically active location: the toxic effect of α-Syn oligomers is enhanced by the presence of monomers. α-Syn oligomers generated by dopamine contain approximately 17 monomers^15^ and may bind multiple Syb2 proteins. Thus, the generation of even a small concentration of α-Syn oligomers may severely inhibit vesicle fusion in presynaptic regions where nonaggregated α-Syn is abundant.

The pathogenesis of PD is heralded by synaptic dysfunction. Impaired vesicular trafficking is thought to occur at the early stages of PD^9,44,45^. A recent study showed that α-Syn oligomers decrease neurotransmitter release and subsequently degenerate nerve terminals prior to cell bodies, implying that PD may break out at the synapse^18^. Our work shows that the α-Syn oligomer interacts with Syb2 to inhibit vesicle fusion. In addition, α-Syn oligomers do not interact with lipid membranes, and thus, the lipid membranes surrounding a cell do not dilute the available α-Syn oligomers. Thus, the low concentration of α-Syn oligomers, which are even assisted by the abundant α-Syn monomer at the presynaptic terminal, can inhibit vesicle fusion very effectively. α-Syn oligomers surrounded by monomers lead to synaptic dysfunction, which may be directly linked to impaired exocytosis of neurotransmitters at the synapse at early stages of PD^46–50^. To reduce the amount of pathological α-Syn, many efforts have been focused on reducing its synthesis or increasing its clearance^19,51–54^. However, α-Syn gene knockdown may hamper the as-yet-unknown physiological functions of α-Syn, and the degree of knockdown required for treatment is largely unexplored^19,51^. Although antibodies against α-Syn have been reported to bind to aggregated forms of α-Syn and exert neuroprotective effects^53,55–57^, there are several hurdles to overcome: crossing the blood–brain barrier, efficient intracellular targeting, inflammation, autoimmune reactions and other side effects^19,51,58^. In addition to these approaches, targeting specific interactions of α-Syn oligomers, which are directly linked to disease progression, can be another therapeutic strategy^59,60^. Our finding that vesicle fusion inhibition can be reversed by small peptides shows that the interaction between Syb2 and α-Syn oligomers can be a potential target to reduce the toxicity of α-Syn oligomers.

## Materials and methods

### Preparation of α-Syn monomer and dopamine-induced oligomers

A gene of recombinant α-Syn fused with glutathione-S-transferase (GST) was clones into a pGEX-KG vector. *Escherichia coli* BL21 Rosetta (DE3) pLysS (Novagene) was used for protein expression. The detailed purification procedures have been described elsewhere^15^. Briefly, the cells were grown at 37 °C in Luria–Bertani (LB) medium with 100 μg/mL ampicillin and induced to express overnight (0.5 mM isopropyl β-d-thiogalactopyranoside) at 16 °C. The harvested cell pellets were resuspended in lysis buffer (1% sarcosine and 2 mM 4-(2-aminoethyl) benzenesulfonyl fluoride hydrochloride in 1 × PBS buffer). After sonication of the cells, the supernatant of the lysate was transferred to a column containing glutathione-agarose beads, which were then incubated for 1.5 h at 4 °C and washed several times with 1 × PBS buffer. A thrombin (30 U) reaction cleaved α-Syn from the resin. For the preparation of α-Syn oligomers, 15 μM α-Syn was incubated with 100 μM dopamine in 20 mM sodium phosphate buffer (pH 7.0) at 37 °C for 72 h. Using size exclusion chromatography using Superdex 200 10/300 GL (GE Healthcare), α-Syn oligomers were purified and concentrated again using an Ultracel 10 K membrane.

### Preparation of SNARE proteins and reconstitution of proteoliposomes

His6×-tagged syntaxin HT (amino acids 168–288; two cysteines replaced with alanines) was cloned into pET28a, and GST-tagged synaptosomal-associated protein25 (SNAP-25) (amino acids 1–206; four cysteines replaced with alanines) and synaptobrevin-2 (amino acids 1–116; one cysteine was replaced with alanine) were cloned into pGEX-KG. Proteins were expressed in *Escherichia coli* BL21 Rosetta (DE3) pLysS (Novagene) cells. The detailed purification procedures have been described elsewhere^61^.

To form liposomes, 1-palmitoyl-2-oleoylsn-glycero-3-phosphocholine (POPC), 1,2-dioleoyl-sn-glycero3-(phospho-l-serine) (DOPS), and cholesterol (Chol) were used (Avanti Polar Lipids). DiI and DiD were used as FRET donor and acceptor dyes, respectively (Invitrogen). The molar ratio POPC:DOPS:Chol:DiI (or DiD) was 71:7:20:2 for both t-vesicles (DiI) and v-vesicles (DiD). SNARE proteins were incorporated into the vesicles at a protein/lipid molar ratio of 1:200. The detailed procedures for preparing proteoliposomes were described elsewhere^32^.

### In vitro lipid-mixing assay

t-Vesicles labeled with DiI and v-vesicles labeled with DiD were mixed together to reach a 20 μM lipid concentration. When α-Syn monomers were treated with α-Syn oligomers, α-Syn monomers were pre-incubated with v-vesicles for 10 min at 37 °C. For the experiments with the peptides, peptides and α-Syn oligomers were mixed with the reaction mixture at the same time (Fig. 5). The final reaction volume was 60 μL. We used 532-nm light for excitation and detected the FRET signal (at 690 nm) every 10 s using a temperature-controlled fluorescence spectrophotometer (Cary Eclipse, Agilent) at 37 °C.

### Western blot

Purified proteins were subjected to 15% SDS/PAGE and then transferred to polyvinylidene fluoride membranes (Bio-Rad), which were then blocked with 5% (w/v) skim milk in Tris-buffered saline with 0.1% Tween 20 detergent (TBST) (500 mM Tris, pH 8.5; 150 mM NaCl; 0.05% Tween 20) for 1 h and incubated with α-Syn primary antibody [sc-52979 (Santa Cruz Biotechnology) at a 1:750 dilution factor] for 12 h at 4 °C. The membrane was rinsed with TBST, incubated with anti-mouse IgG peroxidase secondary antibody (Sigma; 1:2500 dilution factor), washed again with TBST, and reacted with chemiluminescent substrate solution (ThermoFisher Scientific, SuperSignal West Pico Chemiluminescent Substrate) for 1 min. Resulting bands were detected using an LAS-4000 instrument (Fujifilm Life Science).

### Cryo-electron microscopy (cryo-EM)

v-Vesicles were incubated with α-Syn (molar ratio of lipid:α-Syn oligomer: monomer = 200:1:20). A 200-mesh carbon grid (Electron Microscopy Sciences) was glow-discharged using the PELCO easiGlow plasma cleaning system (Ted Pella, Inc.). Sample vitrification was performed using a vitrification robot (FEI Company) by plunging the samples in liquid ethane. A droplet of sample solution (3 μL) was placed on a grid, automatically blotted with filter paper, and plunge-frozen in liquid ethane. The grids were then mounted in dedicated cartridges and stored under liquid nitrogen until data collection. The vitrified specimens were examined using an FEI Talos L120C Cryo-EM instrument (FEI Company) operating at a 120-kV acceleration voltage in the Nanobioimaging center of Seoul National University.

### Fluorescence correlation spectroscopy (FCS)

We incubated v-vesicles doped with 0.02% DiI with/without 10 nM α-Syn oligomers for 10 min at room temperature. We also incubated the v-vesicles with α-Syn T44P/A89P for 10 min, and then α-Syn oligomers were added into the premixture. We performed FCS measurements for each sample for 5 min using a home-built microscope setup^62^, and autocorrelation curves were obtained using a homemade LabVIEW analysis program^32^. Autocorrelation curves were obtained at 100 μM lipid.

### Fluorescence anisotropy measurement

Fluorescence anisotropy measurements were performed using a fluorometer with two linear polarizers (QM-4/2005SE, Photon Technology). 15 nM CFα2 labeled with Cy5 was mixed with various concentrations of the vesicles reconstituted with Syb2 or N-terminal truncated Syb2. Cy5-CFα2 was excited with a polarized light at 633 nm and the emitted light was detected at 650–690 nm through the horizontal and vertical polarizers. Dissociation-equilibrium constants (K_d_) were estimated by a single-site binding equation^63^.

## Supplementary Information

Supplementary Information 1.

## References

[1] Spillantini MG (1997). Alpha-synuclein in Lewy bodies. Nature.

[2] Baba M (1998). Aggregation of alpha-synuclein in Lewy bodies of sporadic Parkinson's disease and dementia with lewy bodies. Am. J. Pathol..

[3] Halliday GM, Holton JL, Revesz T, Dickson DW (2011). Neuropathology underlying clinical variability in patients with synucleinopathies. Acta Neuropathol..

[4] Arawaka S, Saito Y, Murayama S, Mori H (1998). Lewy body in neurodegeneration with brain iron accumulation type 1 is immunoreactive for alpha-synuclein. Neurology.

[5] Wakabayashi K, Matsumoto K, Takayama K, Yoshimoto M, Takahashi H (1997). NACP, a presynaptic protein, immunoreactivity in Lewy bodies in Parkinson's disease. Neurosci. Lett..

[6] Haass C, Selkoe DJ (2007). Soluble protein oligomers in neurodegeneration: Lessons from the Alzheimer's amyloid beta-peptide. Nat. Rev. Mol. Cell Biol..

[7] Conway KA (2000). Acceleration of oligomerization, not fibrillization, is a shared property of both alpha-synuclein mutations linked to early-onset Parkinson's disease: Implications for pathogenesis and therapy. Proc. Natl. Acad. Sci. USA.

[8] Lashuel HA (2002). Alpha-synuclein, especially the Parkinson's disease-associated mutants, forms pore-like annular and tubular protofibrils. J. Mol. Biol..

[9] Lotharius J, Brundin P (2002). Pathogenesis of Parkinson's disease: Dopamine, vesicles and alpha-synuclein. Nat. Rev. Neurosci..

[10] Bucciantini M (2002). Inherent toxicity of aggregates implies a common mechanism for protein misfolding diseases. Nature.

[11] Sharon R (2003). The formation of highly soluble oligomers of alpha-synuclein is regulated by fatty acids and enhanced in Parkinson's disease. Neuron.

[12] El-Agnaf OMA (2006). Detection of oligomeric forms of alpha-synuclein protein in human plasma as a potential biomarker for Parkinson's disease. FASEB J..

[13] Conway KA, Harper JD, Lansbury PT (1998). Accelerated in vitro fibril formation by a mutant alpha-synuclein linked to early-onset Parkinson disease. Nat. Med..

[14] Winner B (2011). In vivo demonstration that alpha-synuclein oligomers are toxic. Proc. Natl. Acad. Sci. USA.

[15] Choi BK (2013). Large alpha-synuclein oligomers inhibit neuronal SNARE-mediated vesicle docking. Proc. Natl. Acad. Sci. USA.

[16] Karpinar DP (2009). Pre-fibrillar alpha-synuclein variants with impaired beta-structure increase neurotoxicity in Parkinson's disease models. EMBO J..

[17] Bengoa-Vergniory N, Roberts RF, Wade-Martins R, Alegre-Abarrategui J (2017). Alpha-synuclein oligomers: A new hope. Acta Neuropathol..

[18] Mor DE (2017). Dopamine induces soluble alpha-synuclein oligomers and nigrostriatal degeneration. Nat. Neurosci..

[19] Dehay B (2015). Targeting alpha-synuclein for treatment of Parkinson's disease: Mechanistic and therapeutic considerations. Lancet Neurol..

[20] Danzer KM (2012). Exosomal cell-to-cell transmission of alpha synuclein oligomers. Mol. Neurodegener..

[21] Buell AK (2014). Solution conditions determine the relative importance of nucleation and growth processes in alpha-synuclein aggregation. Proc. Natl. Acad. Sci. USA.

[22] Giasson BI (2000). Oxidative damage linked to neurodegeneration by selective alpha-synuclein nitration in synucleinopathy lesions. Science.

[23] Uversky VN, Li J, Fink AL (2001). Metal-triggered structural transformations, aggregation, and fibrillation of human alpha-synuclein—a possible molecular link between Parkinson's disease and heavy metal exposure. J. Biol. Chem..

[24] Barnham KJ, Masters CL, Bush AI (2004). Neurodegenerative diseases and oxidative stress. Nat. Rev. Drug Discov..

[25] Burre J, Sharma M, Sudhof TC (2014). Alpha-synuclein assembles into higher-order multimers upon membrane binding to promote SNARE complex formation. Proc. Natl. Acad. Sci. USA.

[26] Burre J (2010). Alpha-synuclein promotes SNARE-complex assembly in vivo and in vitro. Science.

[27] Larsen KE (2006). Alpha-synuclein overexpression in PC12 and chromaffin cells impairs catecholamine release by interfering with a late step in exocytosis. J. Neurosci..

[28] Chandra S, Gallardo G, Fernandez-Chacon R, Schluter OM, Sudhof TC (2005). Alpha-synuclein cooperates with CSP alpha in preventing neurodegeneration. Cell.

[29] Nemani VM (2010). Increased expression of alpha-synuclein reduces neurotransmitter release by inhibiting synaptic vesicle reclustering after endocytosis. Neuron.

[30] Diao JJ (2013). Native alpha-synuclein induces clustering of synaptic-vesicle mimics via binding to phospholipids and synaptobrevin-2/VAMP2. Elife.

[31] Weber T (1998). SNAREpins: Minimal machinery for membrane fusion. Cell.

[32] Kim JY (2012). Solution single-vesicle assay reveals PIP2-mediated sequential actions of synaptotagmin-1 on SNAREs. EMBO J..

[33] Rothman JE (1994). Molecular mechanisms of intracellular protein-transport. J. Neurochem..

[34] Jahn R, Scheller RH (2006). SNAREs—engines for membrane fusion. Nat. Rev. Mol. Cell Biol..

[35] Conway KA, Rochet JC, Bieganski RM, Lansbury PT (2001). Kinetic stabilization of the alpha-synuclein protofibril by a dopamine-alpha-synuclein adduct. Science.

[36] Cappai R (2005). Dopamine promotes alpha-synuclein aggregation into SDS-resistant soluble oligomers via a distinct folding pathway. FASEB J..

[37] Rekas A (2010). The structure of dopamine induced alpha-synuclein oligomers. Eur. Biophys. J. Biophys. Lett..

[38] Sun JC (2019). Functional cooperation of alpha-synuclein and VAMP2 in synaptic vesicle recycling. Proc. Natl. Acad. Sci. USA.

[39] Burre J, Sharma M, Sudhof TC (2015). Definition of a molecular pathway mediating alpha-synuclein neurotoxicity. J. Neurosci..

[40] Elson EL (2011). Fluorescence correlation spectroscopy: Past, present and future. Biophys. J..

[41] Fusco G (2016). Structural basis of synaptic vesicle assembly promoted by alpha-synuclein. Nat. Commun..

[42] Hawk BJD, Khounlo R, Shin YK (2019). Alpha-Synuclein continues to enhance SNARE-dependent vesicle docking at exorbitant concentrations. Front. Neurosci..

[43] Cremades N (2012). Direct observation of the interconversion of normal and toxic forms of alpha-synuclein. Cell.

[44] Hunn BHM, Cragg SJ, Bolam JP, Spillantini MG, Wade-Martins R (2015). Impaired intracellular trafficking defines early Parkinson's disease. Trends Neurosci..

[45] Ebanks K, Lewis PA, Bandopadhyay R (2020). Vesicular dysfunction and the pathogenesis of Parkinson's disease: Clues from genetic studies. Front. Neurosci..

[46] Picconi, B., Piccoli, G. & Calabresi, P. in *Synaptic Plasticity: Dynamics, Development and Disease* Vol. 970 *Advances in Experimental Medicine and Biology* (eds M. R. Kreutz & C. Sala) 553–572 (2012). 10.1007/978-3-7091-0932-8_24 22351072

[47] Volpicelli-Daley LA (2011). Exogenous alpha-synuclein fibrils induce lewy body pathology leading to synaptic dysfunction and neuron death. Neuron.

[48] Kramer ML, Schulz-Schaeffer WJ (2007). Presynaptic alpha-synuclein aggregates, not Lewy bodies, cause neurodegeneration in dementia with Lewy bodies. J. Neurosci..

[49] Diogenes MJ (2012). Extracellular alpha-synuclein oligomers modulate synaptic transmission and impair LTP via NMDA-receptor activation. J. Neurosci..

[50] Schulz-Schaeffer WJ (2010). The synaptic pathology of alpha-synuclein aggregation in dementia with Lewy bodies, Parkinson's disease and Parkinson's disease dementia. Acta Neuropathol..

[51] Olanow CW, Kordower JH (2017). Targeting-synuclein as a therapy for Parkinson's disease: The battle begins. Mov. Disord..

[52] Savitt JM, Dawson VL, Dawson TM (2006). Diagnosis and treatment of Parkinson disease: Molecules to medicine. J. Clin. Investig..

[53] Masliah ERE, Mante M, Crews L, Spencer B, Adame A (2010). Passive immunization reduces behavioral and neuropathological deficits in an alpha-synuclein transgenic model of Lewy body disease. PLoS One.

[54] Forman MS, Trojanowski JQ, Lee VMY (2004). Neurodegenerative diseases: A decade of discoveries paves the way for therapeutic breakthroughs. Nat. Med..

[55] Covell DJ (2017). Novel conformation-selective alpha-synuclein antibodies raised against different in vitro fibril forms show distinct patterns of Lewy pathology in Parkinson's disease. Neuropathol. Appl. Neurobiol..

[56] Masliah E, Rockenstein E, Adame A, Alford M, Crews L, Hashimoto M, Seubert P, Lee M, Goldstein J, Chilcote T, Games D, Schenk D (2005). Effects of α-synuclein immunization in a mouse model of Parkinson’s disease. Neuron.

[57] Mandler M, Valera E, Rockenstein E (2014). Next-generation active immunization approach for synucleinopathies: Implications for Parkinson’s disease clinical trials. Acta Neuropathol..

[58] Brás I, Dominguez-Meijide A, Gerhardt E (2020). Synucleinopathies: Where we are and where we need to go. J. Neurochem..

[59] van Diggelen F (2020). The interactome of stabilized alpha-synuclein oligomers and neuronal proteins. FEBS J..

[60] Betzer C (2015). Identification of synaptosomal proteins binding to monomeric and oligomeric alpha-synuclein. PLoS One.

[61] Kweon DH, Kim CS, Shin YK (2003). Insertion of the membrane-proximal region of the neuronal SNARE coiled coil into the membrane. J. Biol. Chem..

[62] Kim C, Lee OC, Kim JY, Sung W, Lee NK (2015). Dynamic release of bending stress in short dsDNA by formation of a kink and forks. Angew. Chem. Int. Ed..

[63] Marchand JB, Kaiser DA, Pollard TD, Higgs HN (2001). Interaction of WASP/Scar proteins with actin and vertebrate Arp2/3 complex. Nat. Cell Biol..

