# Supplementary material for: Cooperative inhibition of SNARE-mediated vesicle fusion by α-synuclein monomers and oligomers
Source: Sci Rep. 2021 May 26;11:10955. doi: 10.1038/s41598-021-90503-0 (PMC8155056; doi:10.1038/s41598-021-90503-0)
Supplement: Supplementary file 1 — Supplementary Information 1. [file 41598_2021_90503_MOESM1_ESM.docx]

**Supplementary Materials for**

**Cooperative inhibition of SNARE-mediated vesicle fusion by α-synuclein monomers and oligomers**

Gyeongji Yoo^1^, Sanghun Yeou^2^, Jung Bae Son^3^, Yeon-Kyun Shin^4^, and Nam Ki Lee^3,*^

^1^ School of Interdisciplinary Bioscience and Bioengineering, Pohang University of Science and Technology, Pohang 37673, Korea.

^2^ Department of Physics, Pohang University of Science and Technology, Pohang 37673, Korea.

^3^ Department of Chemistry, Seoul National University, Seoul 08826, Korea.

^4^ Roy J. Carver Department of Biochemistry, Biophysics & Molecular Biology, Iowa State University, Ames, Iowa 50011, USA.

*Correspondence to: [namkilee@snu.ac.kr](mailto:namkilee@snu.ac.kr)

**This PDF file includes:**

**Figs. S1 to S12**


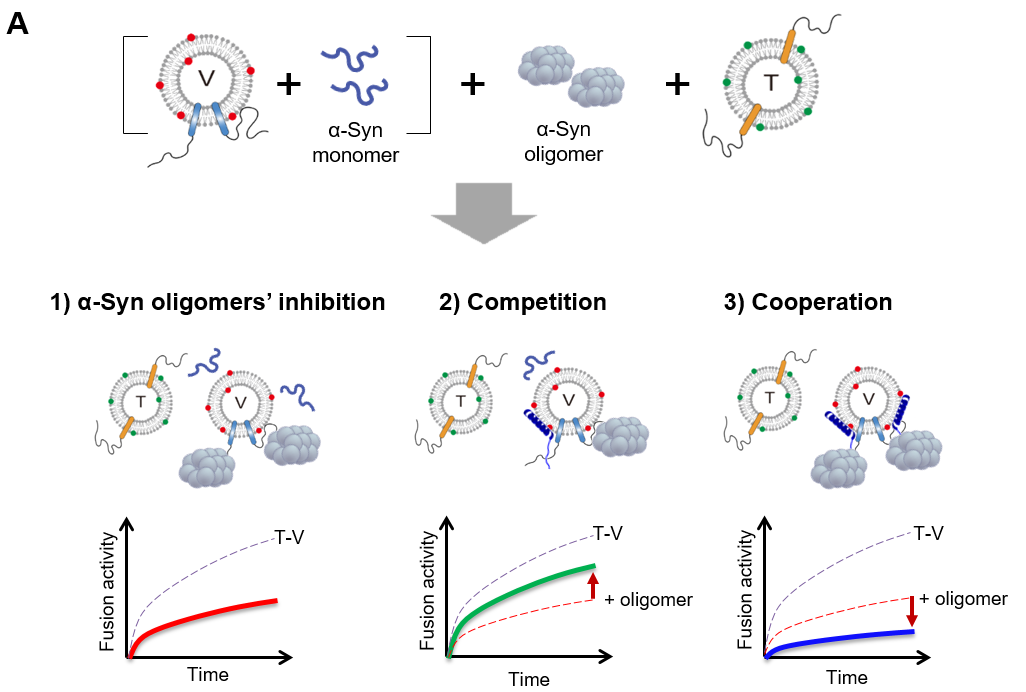


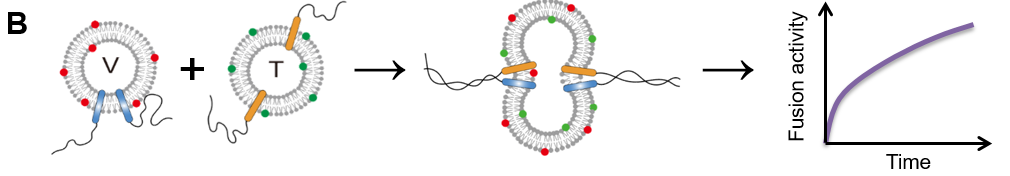


**Fig. S1. Schematic illustration of the possible scenarios when α-Syn monomers and oligomers are mixed with vesicle fusion.**

(A) Three models and the expected FRET results. (1) α-Syn monomer has a weaker binding ability to Syb2 compared with α-Syn oligomers. Thus, α-Syn monomer has no effect on α-Syn oligomers’ fusion inhibitory effect. (2) Because both α-Syn monomer and oligomers commonly interact with Syb2, they compete with each other for Syb2 binding. As a result, the addition of the monomer attenuates the fusion inhibitory effect of oligomers. (3) α-Syn monomer and oligomers cooperate and increase the fusion inhibitory toxicity. (B) Schematic illustration of in vitro vesicle fusion experiment. When t-vesicles doped with donor dyes and v-vesicles doped with acceptor dyes are fused together, lipid mixing occurs and the FRET signal increases. All figures were produced using Microsoft Powerpoint professional plus 2019 (Microsoft, Redmond, Washington, USA) and Adobe Illustrator CS6 (Adobe, San Jose, California, USA) (https://www.adobe.com/kr/products/illustrator.html)


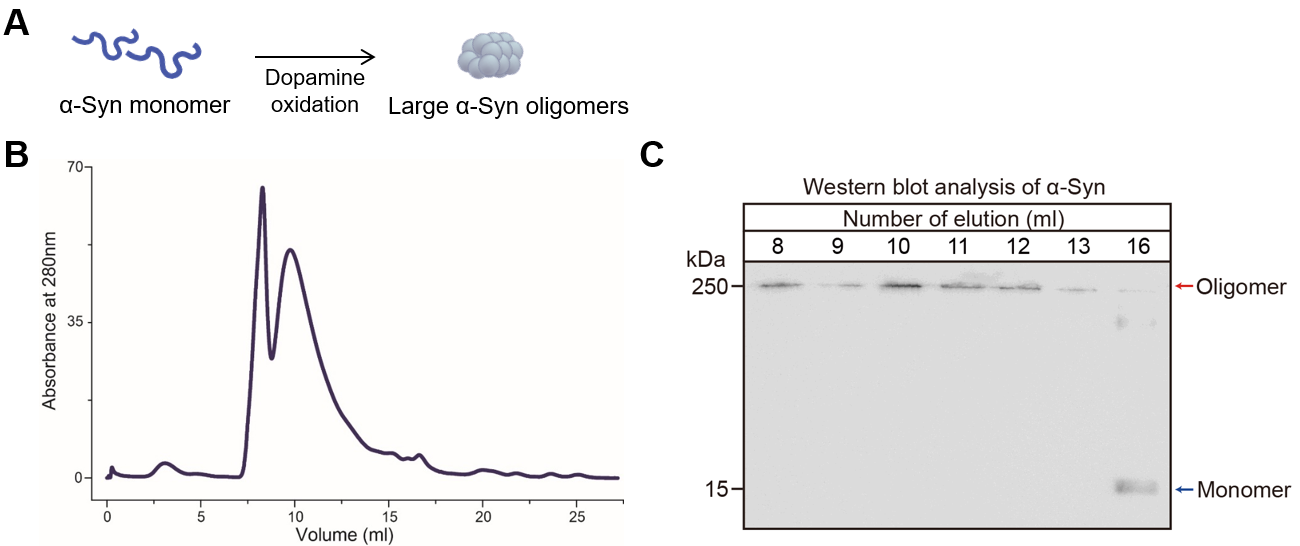


**Fig. S2. Generation and purification of dopamine-induced α-Syn oligomers.**

(A) Generation of α-Syn oligomers by dopamine oxidation. 15 μM α-Syn was incubated with 100 μM dopamine for 72 hours. The figure was created by Adobe Illustrator CS6 (Adobe).

(B) Elution profile of dopamine-induced α-Syn oligomers obtained by the size exclusion chromatography (Superdex 200 10/300 GL column). Each elution fraction (1 mL) was collected in separate tubes. The graph was produced using Originlab 8.5 software (OriginLab Corporation, Northhampton, MA) ([https://www.originlab.com/index.aspx?go= PRODUCTS/Origin](https://www.originlab.com/index.aspx?go=%20PRODUCTS/Origin)) and edited by Adobe Illustrator CS6 (Adobe).

(C) Western blot of the eluted α-Syn oligomers. Each fraction collected from (B) was concentrated and applied to 15% SDS/PAGE. The fractions 8 through 13 contained α-Syn oligomers, whereas the fraction 16 contained mostly α-Syn monomer. The image was edited by Adobe Illustrator CS6 (Adobe).


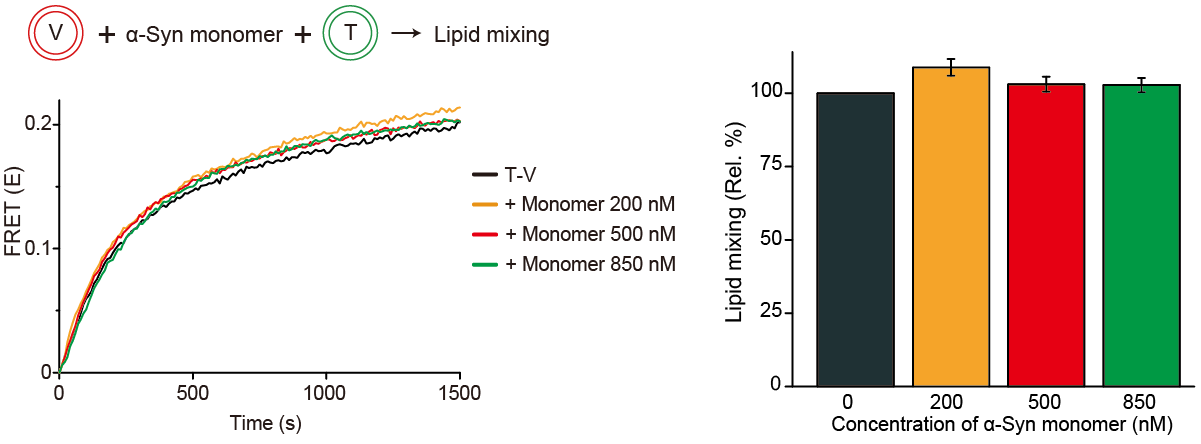


**Fig. S3. α-Syn monomer at submicromolar concentrations has no effect on SNARE-mediated lipid mixing.**

v-Vesicles were incubated with α-Syn monomer at the concentrations from 200 nM to 850 nM for 10 min and then t-vesicles were added. α-Syn monomer showed a negligible effect on SNARE-mediated vesicle fusion (±S.E.M., N=3). The graphs shown were produced using Originlab 8.5 software (OriginLab Corporation) and edited by Adobe Illustrator CS6 (Adobe).


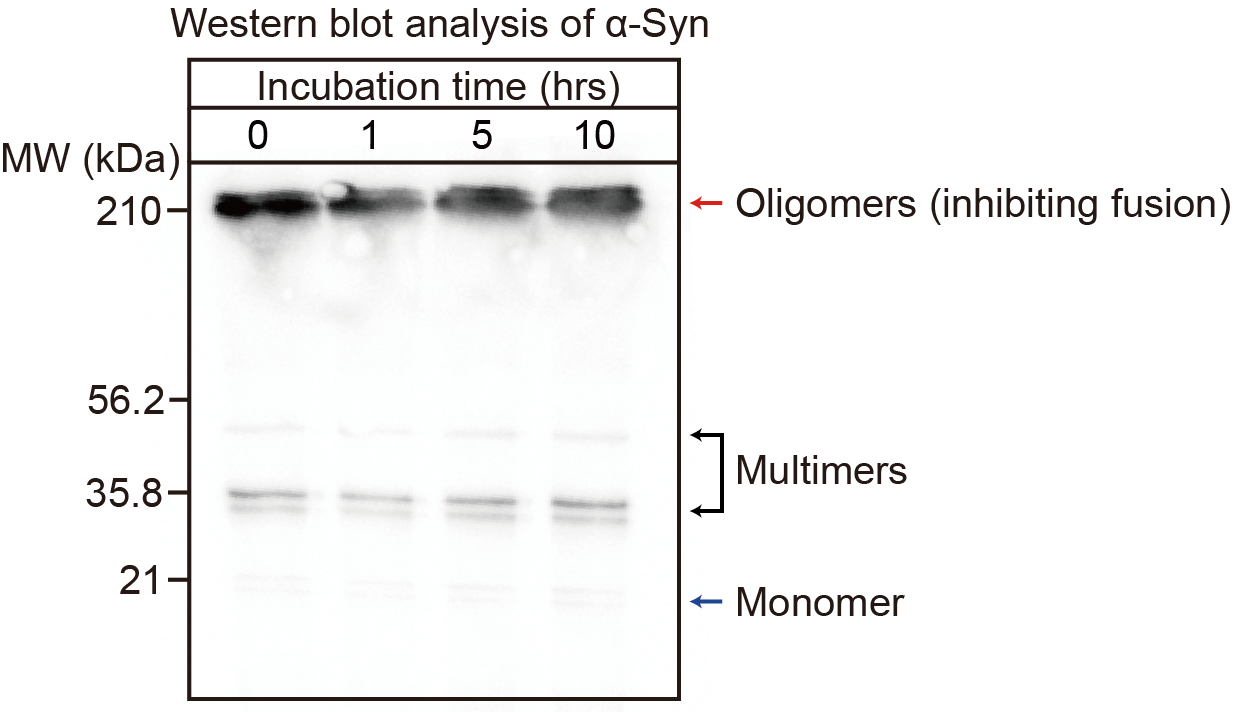


**Fig. S4. Incubation of α-Syn monomers with dopamine-induced α-Syn oligomers in the presence of vesicles****.** Vesicle compositions are 73% PC, 20% cholesterol, and 7% PS. α-Syn monomers (500 nM) were incubated with the purified dopamine-induced oligomers (10 nM) at 37 ℃ for up to 10 hours and then the mixtures were applied to the Western blot analysis and probed with anti–α-Syn antibody. No change in the intensities of the oligomers, multimers, and monomer was observed. The image was edited by Adobe Illustrator CS6 (Adobe).


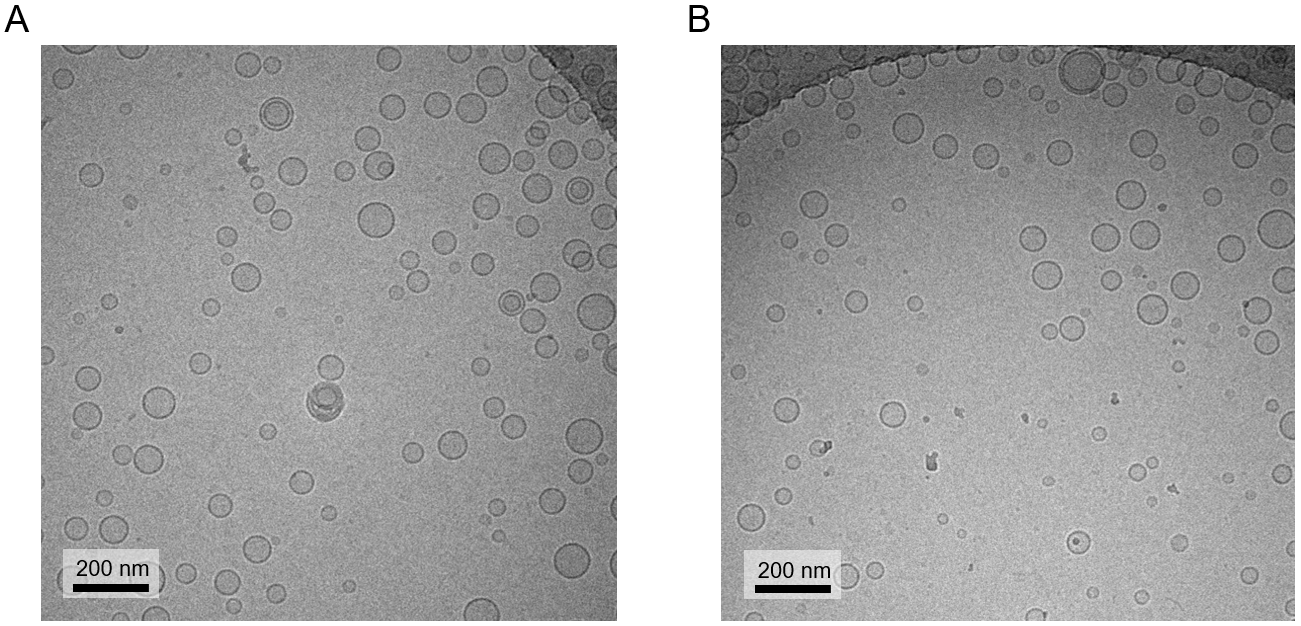


Fig. S5. Cryo-EM images of vesicles incubated with α-Syn oligomers.

(A) Vesicles (5 μM in lipid concentration) reconstituted with the N-terminal truncated synaptobrevin-2 were incubated with α-Syn oligomers (25 nM) at room temperature for 10 min. (B) Protein-free vesicles in the presence of α-Syn oligomers. No clustering of vesicles was observed in both cases. The images were edited by Microsoft Powerpoint professional plus 2019 (Microsoft).


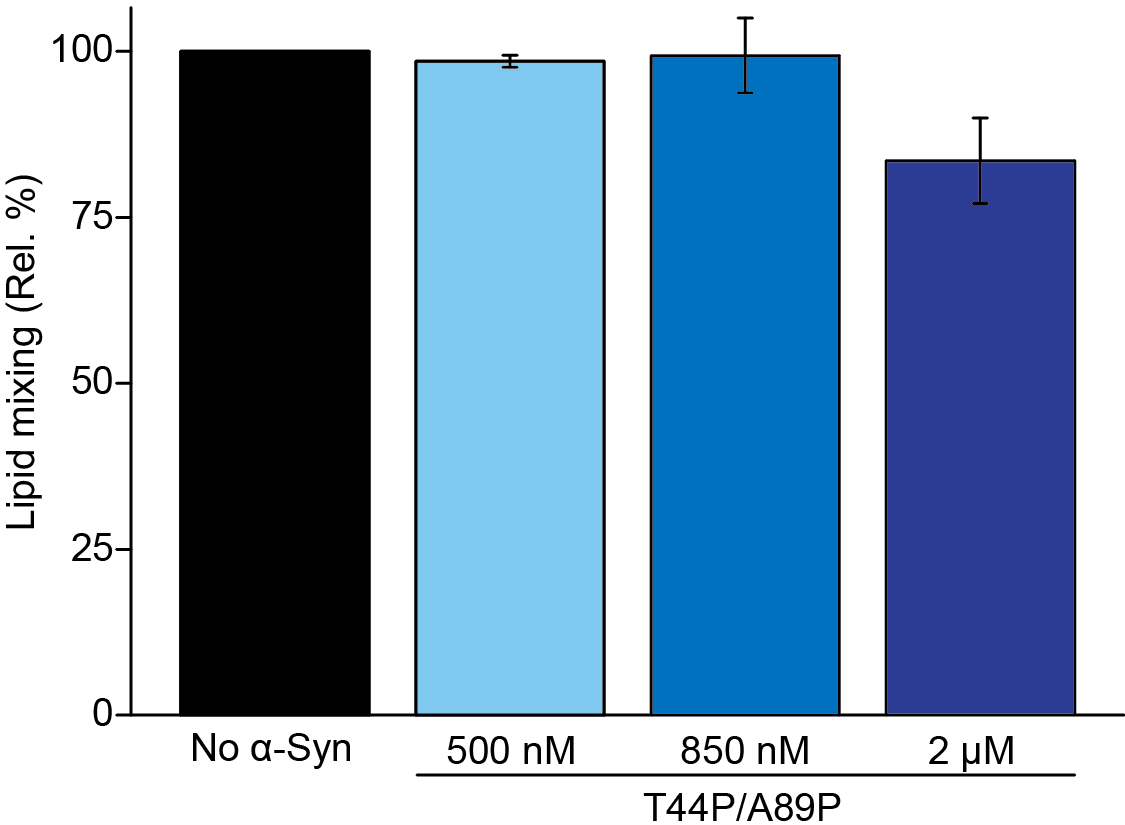


**Fig. S6. Effect of the T44P/A89P monomer on SNARE-mediated vesicle fusion.**

The T44P/A89P α-Syn monomer was incubated with v-vesicles (no α-Syn oligomers), and then t-vesicles were added to the premixture. The T44P/A89P monomer has no effect on the vesicle fusion up to 850 nM, but approximately 82% lipid mixing was observed with 2 μM of T44P/A89P (±S.E.M., N=3). The graph was produced using Originlab 8.5 software (OriginLab Corporation) and edited by Adobe Illustrator CS6 (Adobe).


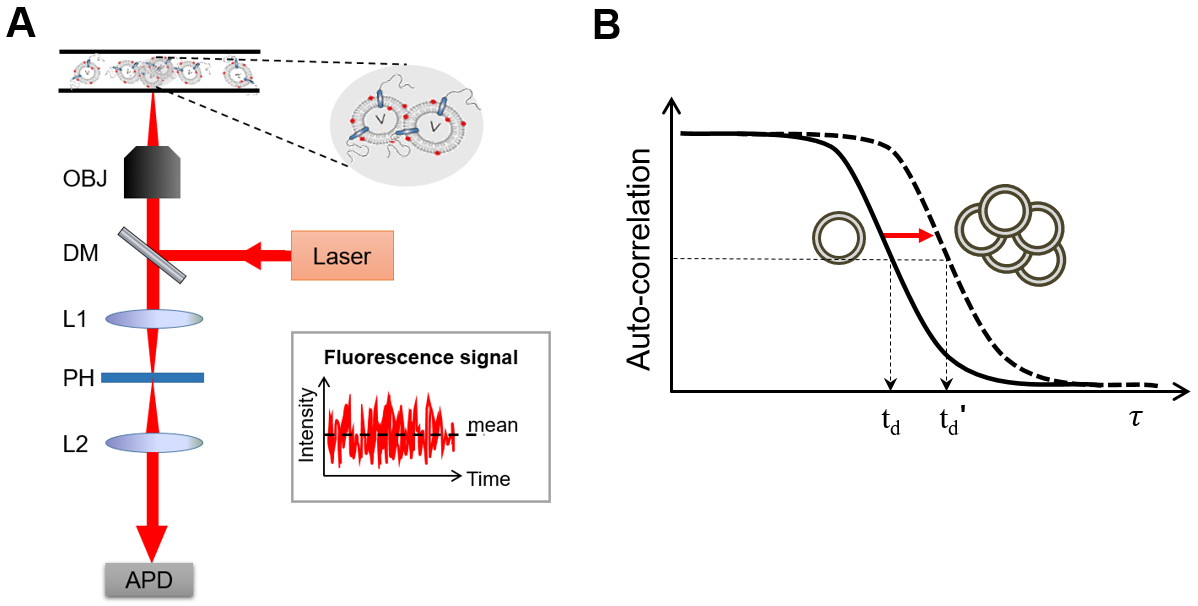


Fig. S7. Fluorescence correlation spectroscopy measurements for v-vesicle clustering induced by α-Syn.

(A) The experimental set-up for fluorescence correlation spectroscopy (FCS) measurements used in this work. OBJ: Objective lens, DM: Dichroic mirror, L: Lens, PH: Pinhole, APD: Avalanche photodiode.

(B) Schematic illustration of autocorrelation curves depending on the size of vesicle clusters. FCS measures the transit time or diffusion time (t_d_ = τ at G = 0.5) of vesicles through the confocal volume. Vesicle clusters (the dotted line) diffuse slower than unclustered vesicles (the solid line) in solution. Thus, the diffusion time of vesicle clusters (t_d_’) is larger than that of unclustered vesicles (t_d_). The figures shown were created by Adobe illustrator CS6 (Adobe) and Microsoft Powerpoint professional plus 2019 (Microsoft).


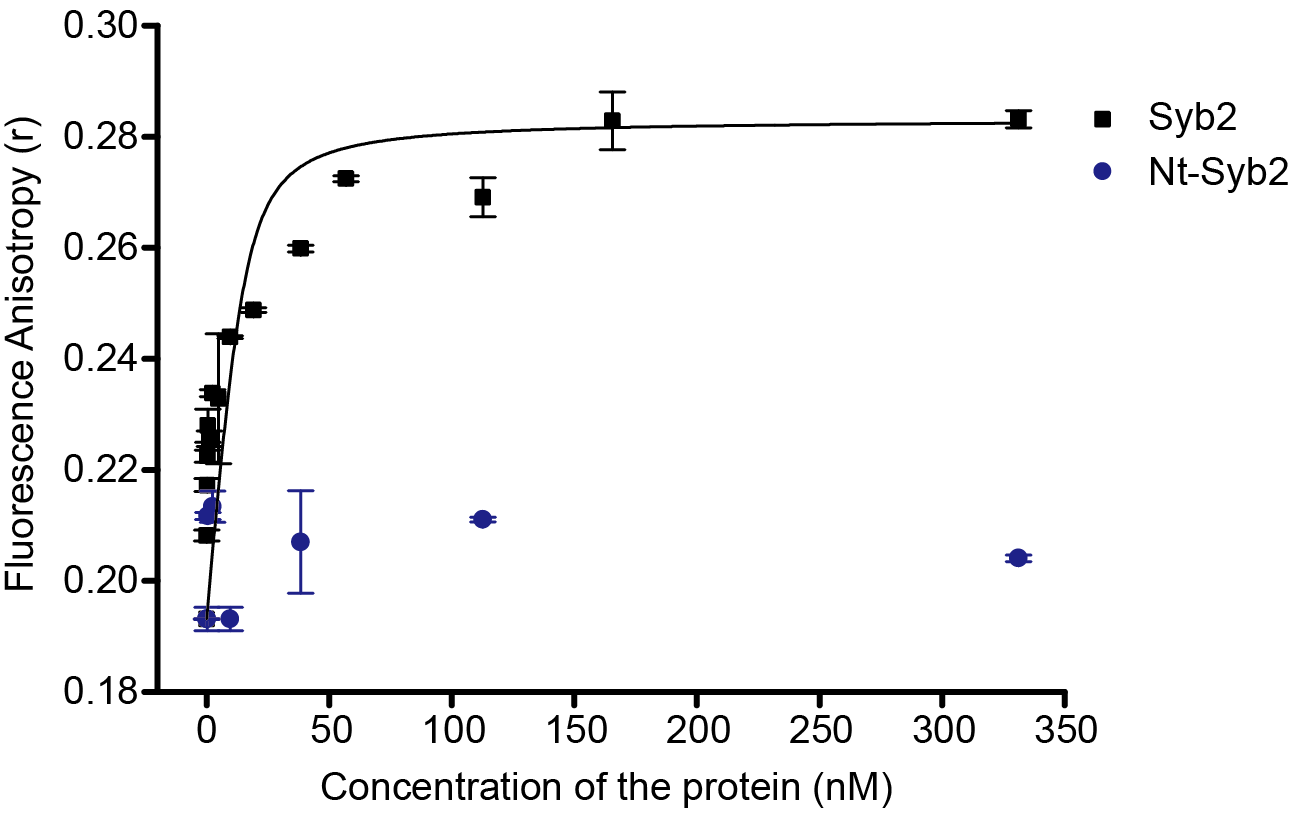


Fig. S8. Fluorescence anisotropy assay for the binding between the CFα2 peptide and synaptobrevin-2 (Syb2). CFα2 (96-125 aa of α-Syn) was labelled with Cy5. We used 15 nM Cy5-CFα2 and increased the concentration of vesicles with full-length Syb2 (the black squares). The concentration of Syb2 in x-axis was calculated from the molar ratio of the lipid to protein. By fitting the curve using a single-site binding equation, we obtained approximately 3 nM Kd. In contrast, when vesicles with N-terminal truncated Syb2 were added, the fluorescence anisotropy of Cy5-CFα2 was nearly invariant (the blue circles). This result confirms that the peptide binds to the N-terminus of Syb2. The graph was produced using Originlab 8.5 software (OriginLab Corporation) and edited by Adobe Illustrator CS6 (Adobe).


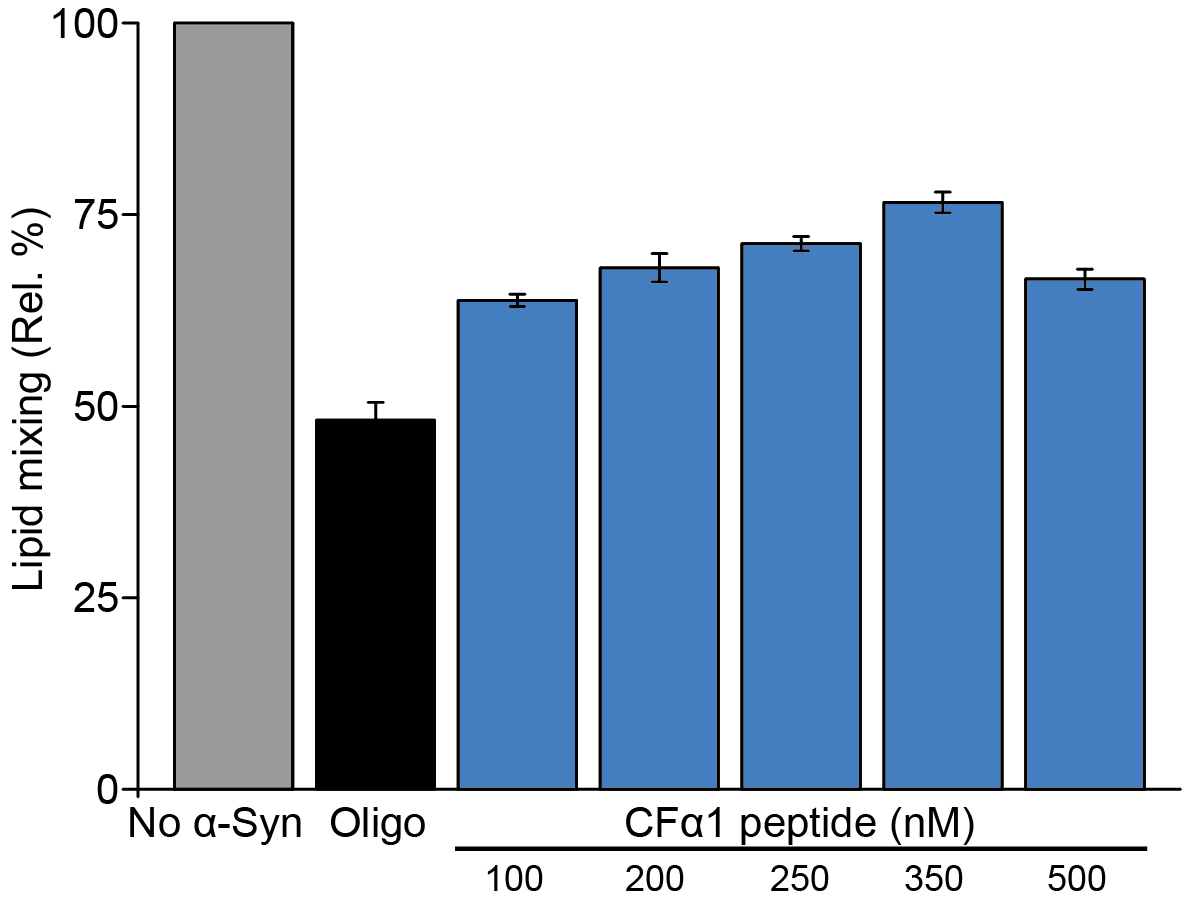


**Fig. S9. The reversal of the fusion inhibition of α-Syn oligomers by CFα1.** CFα1 peptide reverses the fusion inhibitory effect of α-Syn oligomers (10 nM). 350 nM CFα1 increased the fusion efficiency to 75% (±S.E.M., N=3). The graph was produced using Originlab 8.5 software (OriginLab Corporation) and edited by Adobe Illustrator CS6 (Adobe).


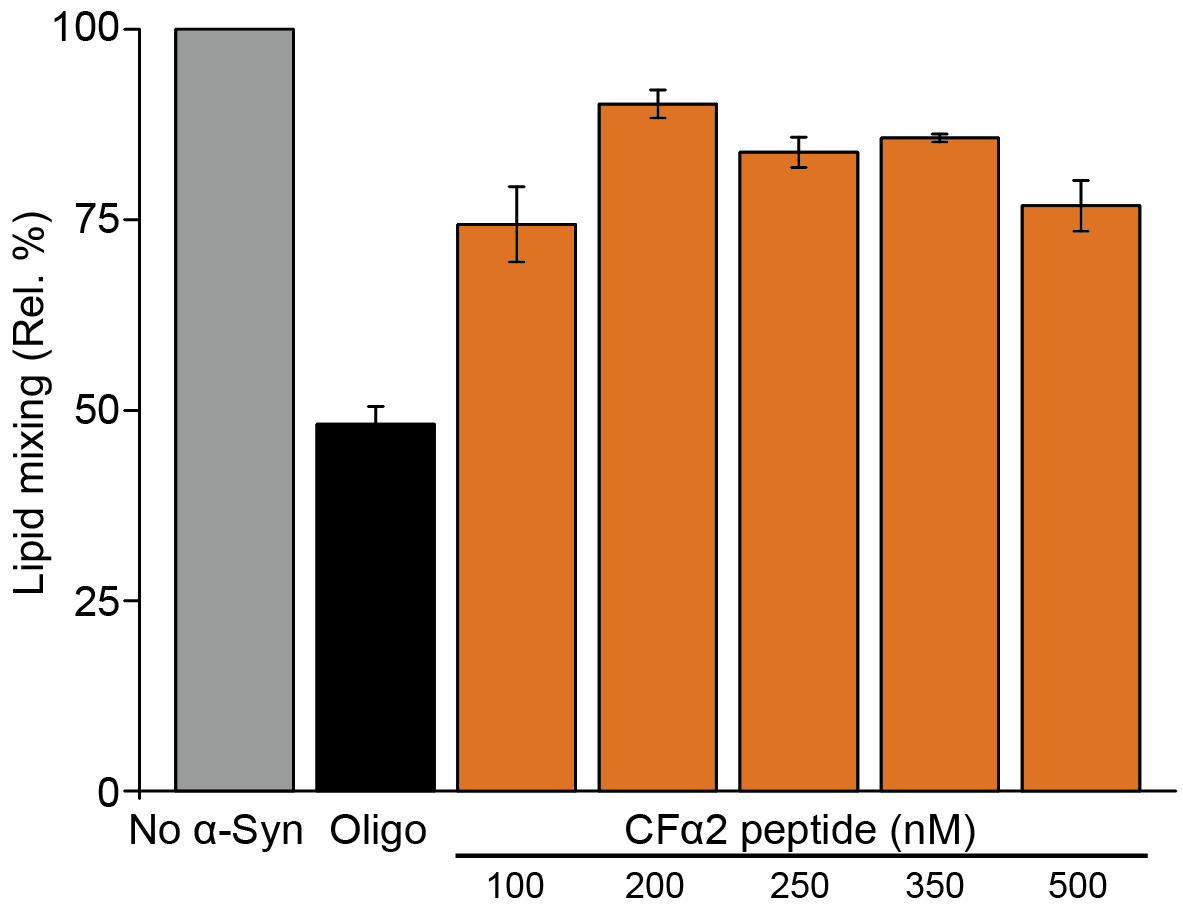


**Fig. S10. The reversal of the fusion inhibition of α-Syn oligomers by CFα2.** 200 nM CFα2 increased the fusion efficiency to 90% (±S.E.M., N=3). The graph was produced using Originlab 8.5 software (OriginLab Corporation) and edited by Adobe Illustrator CS6 (Adobe).


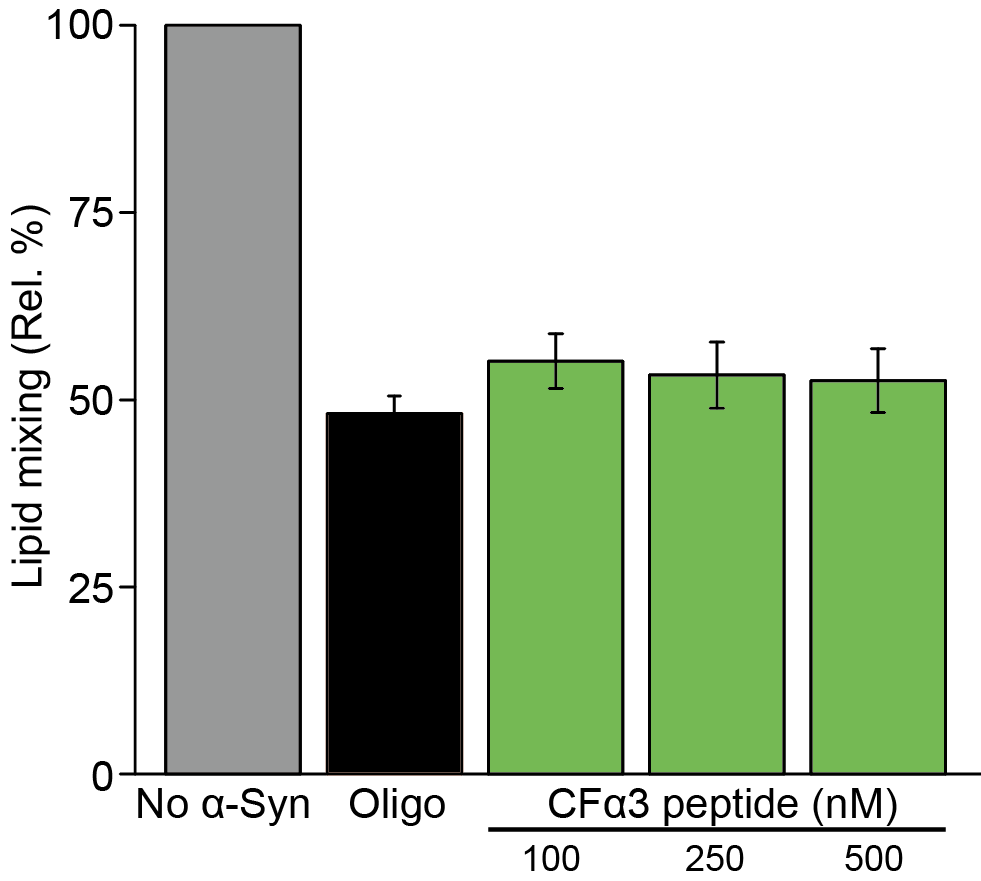


**Fig. S11. The reversal of the fusion inhibition of α-Syn oligomers by CFα3.** CFα3 had no effect on the reversal of the fusion inhibition (±S.E.M., N=3). The graph was produced using Originlab 8.5 software (OriginLab Corporation) and edited by Adobe Illustrator CS6 (Adobe).


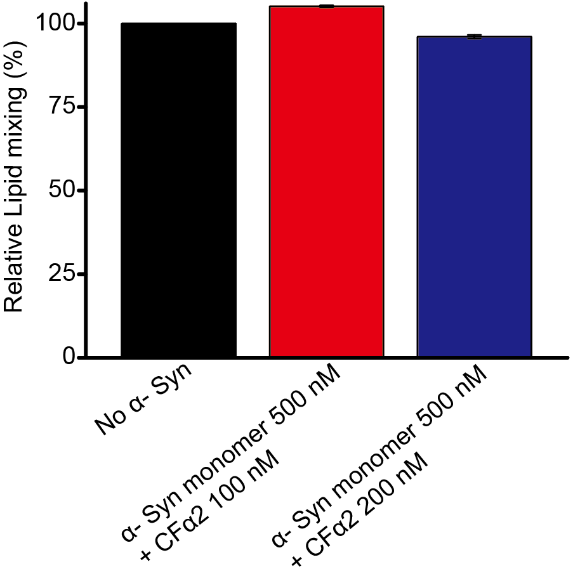


Fig. S12. The effect of the peptide on SNARE-mediated vesicle fusion in the presence of α-Syn monomers. For a lipid mixing assay, we added 500 nM monomeric α-Syn together with CFα2 peptides. CFα2 peptide showed a negligible effect on SNARE-dependent vesicle fusion in the presence of α-Syn monomers (±S.E.M., N=3). The graph was produced using Originlab 8.5 software (OriginLab Corporation) and edited by Adobe Illustrator CS6 (Adobe).
